# A Family of Algorithms for Computing Consensus about Node State from Network Data

**DOI:** 10.1371/journal.pcbi.1003109

**Published:** 2013-07-18

**Authors:** Eleanor R. Brush, David C. Krakauer, Jessica C. Flack

**Affiliations:** 1 Program in Quantitative and Computational Biology, Princeton University, Princeton, New Jersey, United States of America; 2 Center for Complexity and Collective Computation, Wisconsin Institute for Discovery, Madison, Wisconsin, United States of America; 3 Santa Fe Institute, Santa Fe, New Mexico, United States of America; 4 Department of Genetics, University of Wisconsin Madison, Madison, Wisconsin, United States of America; University of Chicago, United States of America

## Abstract

Biological and social networks are composed of heterogeneous nodes that contribute differentially to network structure and function. A number of algorithms have been developed to measure this variation. These algorithms have proven useful for applications that require assigning scores to individual nodes–from ranking websites to determining critical species in ecosystems–yet the mechanistic basis for why they produce good rankings remains poorly understood. We show that a unifying property of these algorithms is that they quantify consensus in the network about a node's state or capacity to perform a function. The algorithms capture consensus by either taking into account the number of a target node's direct connections, and, when the edges are weighted, the uniformity of its weighted in-degree distribution (*breadth*), or by measuring net flow into a target node (*depth*). Using data from communication, social, and biological networks we find that that *how* an algorithm measures consensus–through breadth or depth– impacts its ability to correctly score nodes. We also observe variation in sensitivity to *source biases* in interaction/adjacency matrices: errors arising from systematic error at the node level or direct manipulation of network connectivity by nodes. Our results indicate that the breadth algorithms, which are derived from information theory, correctly score nodes (assessed using independent data) and are robust to errors. However, in cases where nodes “form opinions” about other nodes using indirect information, like reputation, depth algorithms, like Eigenvector Centrality, are required. One caveat is that Eigenvector Centrality is not robust to error unless the network is transitive or assortative. In these cases the network structure allows the depth algorithms to effectively capture breadth as well as depth. Finally, we discuss the algorithms' cognitive and computational demands. This is an important consideration in systems in which individuals use the collective opinions of others to make decisions.

## Author Summary

Decision making in complex societies requires that individuals be aware of the group's collective opinions about themselves and their peers. In previous work, social power, defined as the consensus about an individual's ability to win fights, was shown to affect decisions about conflict intervention. We develop methods for measuring the consensus in a group about individuals' states, and extend our analyses to genetic and cultural networks. Our results indicate that *breadth* algorithms, which measure consensus by taking into account the number and uniformity of an individual's direct connections, correctly predict an individual's function even when some of the group members have erred in their assessments. However, in cases where nodes “form opinions” about other nodes using indirect information algorithms that measure the *depth* of consensus, like Eigenvector Centrality, are required. One caveat is that Eigenvector Centrality is not robust to error unless the network is transitive or assortative. We also discuss the algorithms' cognitive and computational demands. These are important considerations in systems in which individuals use the collective opinions of others to make decisions. Finally, we discuss the implications for the emergence of social structure.

## Introduction

A goal of many of network studies (*e.g.* [1]–[4]) is to predict the effects of perturbations, such as extinction and predation events, on network structure. Making these predictions requires information about network connectivity (*e.g.* is the network scale-free, exponential, etc.). When the connectivity is non-uniform, it is also important to quantify variation at the node level in order to identify nodes that, if removed, are likely to impact negatively network stability. This is well recognized and many useful methods have been developed for measuring this variation [1]–[2], [5]–[14] in a range of networks, including the world-wide web [5], food webs describing trophic interactions [1], [2], networks of interactions between genes and proteins [6]–[10], and social networks, in both animal and human societies [11]–[16].

Patterns of connectivity can also influence node function in the larger system of which the network is a part. For example, in previous work on the behavioral causes of multi-scale social structure in primate societies [14], [17]–[22] it was found that group consensus about an individual's ability to win fights – its social power (see Sec. Primate communication network)–is population coded in a status signaling network. In this system, individuals use subordination signals to communicate to adversaries that they perceive themselves to be the weaker opponent. The signals are often repeated and are always unidirectional (emitted by one individual in a pair but not the other). A single signal indicates that the sender perceives the receiver capable of using force successfully against him. The frequency of signals emitted (over some defined time period) indicates the strength of the sender's perception that the receiver can successfully use force against him. In the work cited above it was demonstrated that consensus in the group about individual 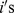 ability to successfully win its fights can be calculated by quantifying uniformity in the weighted in-degree distribution of signals sent to 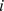 by its senders and weighting this score by the total number of signals 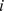 received (this calculation is described in Sec. Shannon consensus). The resulting score for 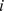 may not be the preferred score for 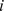 of any specific group member, but can be said to reflect the group's collective view about how good 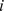 is at winning fights. Correspondingly, the rank order associated with the distribution of scores in the population might not match the preferred rank order of any single individual, but as the outcome of integrating over all of the individual opinions, it can be said to be the consensus social power rank order.

The data indicate that individuals can estimate their own social power and also know something about how others in the group are collectively perceived [17], [20], [23], [24]. Consequently, social power is informative about the likely cost of interaction when interactions are not strictly pair-wise (a common feature of these systems and the reason why a consensus-based definition is important) [17]–[19]. Under heavy-tailed power distributions, in which a few individuals are disproportionately powerful, conflict management mechanisms like third-party policing (a critical social function) can emerge and are performed by nodes in the tail of the power distribution [14], [20]. Policing is an important social function because by controlling conflict it facilitates edge building by nodes in the signaling as well as other social networks [18], [20]. These results suggest that (1) network structure can encode node function and that (2) measures that quantify agreement in node connectivity patterns can be used to decode this population coding of node function. In Table 1 we give several examples of other networks in which node function might also be population coded and consensus estimation could be useful for identifying important nodes.

**Table 1:** The interpretation of consensus about the state of a node, or its capacity to perform a behavior, depends on the type of interactions constituting edges in the network.

Interaction	Node State	Functional Consequences
Subordination signal	Social power	Conflict management behavior
Collaboration	Scientific reputation	Grants awarded
Functional linkage	Gene importance	Growth/Fitness
Friendship	Popularity	Friends/Gifts
Citation	Influence	Grants/Positions
Trade	Quality of goods	Prevalence of goods

We suggest and find that the consensus about this state predicts function. This result is strongest for the subordination signaling network, for which the mechanistic basis of consensus is best understood and the data strongly indicate that the subordination signals are not proxies for power but are direct measures of it.

In principle, consensus about node state or function can be quantified by measuring the uniformity of a node's weighted in-degree distribution [14], as in the above example, by measuring the “flow” into and out of a node (depth), or using simple counts. To capture these competing notions of consensus, we introduce a variety of alternative information theoretic, diffusion, and count algorithms that capture breadth and depth to different degrees, and so serve as hypotheses about how functional variation in nodes is encoded in interaction networks via consensus. The algorithms take an interaction network as input and produce a vector of scores for the nodes in the network as output. We interpret the score of node 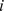 as the collective opinion, or consensus, about 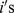 state or its capacity to perform a given behavior. We note that the algorithms only quantify agreement in the connectivity patterns; what the consensus is about– 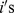 state– depends on the type of interactions in the interaction network. For example, in the work on power in primate societies mentioned above, the interaction matrix contained directed subordination signals. These signals have special properties that allow them to reliably encode information about the ability to win fights, which is the basis of power [19]. We discuss the importance of the interaction matrix for the interpretation of consensus in greater detail in Sec. Background and motivation.

After introducing the algorithms, we compare their mathematical properties, and in a few cases, establish approximate equivalence. We introduce three data sets that we use to empirically evaluate how well the output of the algorithms predicts node function out of sample. We investigate the properties of these algorithms that make them predictive measures of consensus. The data sets include a status communication network in a primate society, a network of collaborating condensed matter physicists from a prominent journal, and a functional linkage network of yeast genes that influence viability and growth. Finally, we assess the sensitivity of the algorithms to systematic error at the node level and strategic manipulation of the network by nodes or small sub-sets of the network.

## Results

For all of the algorithms we consider, we begin with a matrix of interactions 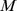. (For discussion of data in the interaction matrix, see Secs. Primate communication network, Physics collaboration network, and Functional gene linkage network). We adopt the convention that if the interactions are directed 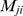, denotes the number of interactions traveling from individual 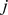 to individual 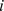. Tables 2 and 3 contain alphabetical lists of all matrices and variables used in the text.

**Table 2:** Matrices used in the text.

Name	Entries	Definition
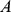	redistribution probabilities used for Eigenvector Centrality	we use 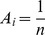
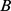	binary interaction matrix	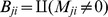
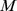	interaction matrix	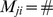 of interactions from 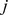 to 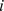
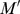	shuffled interaction matrix	
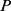	probabilities of direction of an interaction used for David's Score	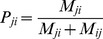
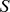	column stochastic matrix used for Shannon Consensus	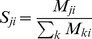
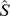	modified version of 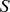 used for Shannon Consensus	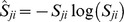
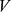	votes used for Borda Count	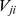 = rank given node 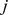 by node 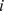
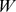	row stochastic matrix used for Eigenvector Centrality	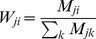
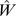	modified version of 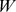 for Eigenvector Centrality	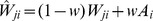

**Table 3:** Variables used in the text.

Variable	Definition
*β_i_*	*Borda Count*
*C_i_*	*Eigenvector Centrality* with uniform redistribution
*D_i_*	*Simple Consensus* (number of partners), 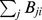
Δ*_i_*	*Weighted Simple Consensus*, 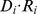
*DS_i_*	*David's Score*
*H_i_*	Shannon entropy, 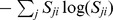
*GL_i_*	*Graph Laplacian*
Π*_i_*	*Shannon Consensus*, 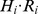
*R_i_*	weighted in-degree, or number of interactions received, 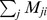

In the text, the subscript *i* on the algorithms is sometimes omitted, in which case the variable refers to the vector of scores rather than a node's score.

The algorithms we compare fall into three classes with respect to consensus: algorithms that accord higher scores to nodes with a more uniform in-degree distribution (breadth algorithms), algorithms that accord higher scores to nodes that are sinks in the network (depth algorithms), and count based algorithms, like the Borda count. For each algorithm, we construct a new matrix that gives the strength of the interactions in a way that depends on what the algorithm is meant to measure. For the breadth algorithms, we calculate the strength of the interaction between nodes 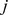 and 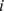 in a way that depends on all of 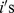 in-edges and reflects the uniformity of the interactions from the whole population to 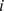. These measures therefore measure the *breadth* of consensus.

We then perform matrix operations on the appropriately transformed interaction matrix. The depth algorithms treat events as a diffusion process over the network and weight more heavily interactions with partners who themselves have many interaction partners and so forth. To capture this chain-like property of interactions, we do repeated matrix operations. These measures therefore measure the *depth* of consensus. We can quantify depth by the number of matrix operations required to perform the algorithm or, equivalently, the length of the “chains” in the network that affect the algorithm. Figure S1 shows a flow chart that summarizes the steps required to derive the distribution of consensus scores under each algorithm. We discuss the computational complexity of the algorithms in Section Cognitive and computational complexity and in the Text S1, Section Computational complexity).

### Breadth algorithms

#### Simple Consensus

The simplest measure of the breadth of the interactions to a given node is the number of nodes that send it interactions. To calculate this, we create a binary matrix 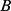, where 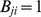 if 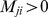 and 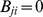 otherwise, and then take the sum, 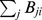. We write 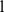 for a column vector of all ones and define the vector
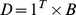
so that 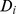 is the number of nodes who send interactions to the target node, 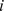. We call this measure *Simple Consensus*.

#### Weighted Simple Consensus

*Weighted Simple Consensus* takes into account the breadth of the interactions flowing into 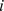 and the total frequency of 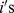 interactions, capturing the “magnitude” of agreement. Total frequency is taken into account as well as uniformity because we want to be able to distinguish a node that has received ten interactions (*e.g.* signals) from each of ten other nodes per unit time from a node that has received only one interaction from each of ten nodes per unit time [14]. We define the vector
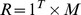
so that 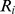 is the weighted in-degree or number of interactions sent to the target node 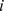. We then define
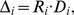
which is the Weighted Simple Consensus score for node 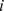.

#### Shannon Consensus

*Shannon Consensus*, like Weighted Simple Agreement, multiplies the uniformity of interactions by the frequency of interactions or weighted in-degree (this measure was introduced in [14], where it was called the Social Power Index). In this case, the uniformity of the interaction weights is quantified using Shannon entropy. Shannon entropy has the desirable property that it is minimized (and in fact equals 0) when there is only one partner, is maximized when all partners send the same number of interactions (*e.g.* signals), and is an increasing function of the number of partners. These properties agree with our intuitions about consensus for the following reasons. When only one node has an opinion consensus is not a meaningful concept, consensus is maximal when everyone has the same opinion, and the amount of consensus possible should increase as the number of nodes involved increases (see Text S1, Section Sample entropy issues, for discussion of sample entropy issues) [14].

Formally, we create a column stochastic interaction matrix 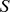 such that 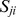 is the probability of one of 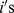 interaction flowing from node 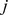, 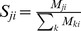. The Shannon entropy of this distribution is 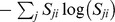. If we define the matrix 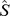 such that 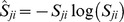 then we can write the vector of the entropies of the interactions received by each node as
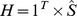
We then define
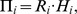
which is the Shannon Consensus score for node 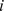. Shannon Consensus thus accords high scores to nodes who receive many interactions *and* who receive similar numbers of interactions from each of their partners.

Given that the entropy of interactions received increases with the number of interaction partners (the maximum entropy is in fact equal to the logarithm of the number of interaction partners), interaction partner number, 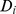, gives a rough estimate of the entropy of interactions received. 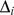 can therefore be thought of as a cognitively and computationally simpler version of Shannon Consensus.

#### Note

In previous work Flack and Krakauer [14] determined whether Shannon entropy alone, 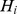, is predictive of consensus with respect to social power. It was found to be a poor predictor of functional data as it can not distinguish between nodes in networks with uniform in-degree differing in the amplitude of their edge weights. Flack and Krakauer also considered total frequency of interactions (in that case, subordination signals) received by a target node, the weighted in-degree, or 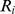, as a potential power measure, but found this to be a poor predictor after controlling for the degree of correlation between frequency and entropy present in the data set (individuals who received many signals also received them from many individuals). We discuss this issue, which relates to the issue of sensitivity to source bias, in detail in Section sensitivity of the algorithms to source biases, and we discuss related count algorithms in Section Count algorithms.

### Depth algorithms

#### Eigenvector Centrality

Unlike with the breadth algorithms, where we consider only interactions directly between 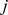 and 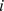, interactions now *bear the trace of the interactions that node* 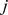 *had with its neighbors*. By weighting the contributions of partners by their own history of interactions, we allow for non-local effects. We can capture these effects with the well-known algorithm *Eigenvector Centrality*. This algorithm has previously been used to measure authority or importance in networks ranging from food webs to networks of websites to citation networks [1], [2], [5], [25].

We now turn 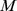 into a row stochastic matrix 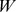 where 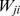 is the number of edges from node 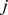 to node 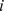, normalized by the total number of edges coming from node 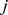, and thus represents the probability of one of node 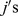 interactions flowing to node 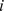. In our notation, 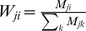. If 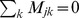 (i.e. node 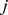 sends no interactions), we set 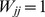 and 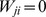 for 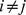. In defining Shannon entropy, we defined a column stochastic matrix 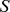 where 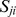 represents the probability of one of 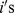 interactions flowing from node 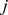 so that we could measure how uniformly the population sends interactions to 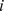. Here we use the row stochastic matrix 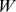 so that we can measure the strength of the flow out from 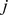 to 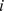.

A node in a graph should be considered more important if it is central, that is, many other important nodes connect to it. Thus, the Eigenvector Centrality of a node 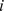 is the sum of centralities of the nodes 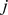 that connect to it weighted by the strength of the connection from node 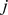 to 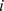. A problem arises in this calculation if there are nodes 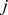 that have no out-edges (in which case, 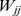 is set to 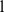): centrality will accumulate on these nodes and the centrality scores corresponding to the nodes that *do* have out-edges, will be 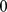. Eigenvector Centrality will be uninformative about the differences in centrality between nodes that do have out-edges. Such absorbing states are likely to be present in many data sets. For example, in the primate group (see Sec. Background and motivation), there is one individual who does not emit any subordination signals and who will thus be an absorbing state. (As discussed in Sec. Background and motivation, the monkeys only signal if they perceive the receiver to be better able to win fights. Additionally, the signals are unidirectional such that if 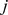 signals to 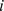, 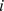 will not signal back to 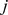 while the dominance relationship is stable.)

One solution is to provide a vector 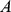 that adds some amount of centrality to each node, with the condition that 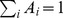 [2], [5]. If we define the matrix 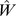 so that 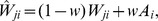 we can define the vector 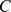 as the solution to 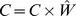 where 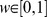. Equivalently,
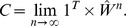


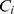
 is then the Eigenvector Centrality score for node 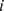. Eigenvector Centrality has infinite depth since it is calculated by multiplying 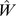 with itself infinitely many times.

We use uniform redistribution probabilities, i.e. 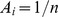 for all 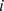, as in [2], [5]. In the case of the primate communication network, we considered several “socially principled” mechanisms for redistribution, including various metrics computed using independently collected affiliation data. These methods for redistribution required additional computational steps and did not improve prediction, so we do not present them here. We also have to specify a redistribution weight 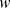. For each test system, we find the redistribution weight 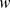 that maximizes the predictive value of the Eigenvector Centrality scores (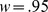 for the primate communication and scientific collaboration networks and 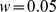 for the functional linkage network). We give further details and discuss how to choose redistribution probabilities when independent functional data is not available in the Supplementary Information (Section Empirical evaluation of redistribution weights for Eigenvector Centrality).

#### David's Score

Another algorithm in the diffusion category is *David's Score*, a measure that has previously been used to generate dominance hierarchies in animal societies [26]–[29]. Here, we use the observed interaction frequencies to calculate the probabilities of interactions (*e.g.* signals in the case of the primate network) being given or received between each pair of nodes. Formally, we define the matrix 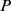 such that 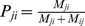 and then define the vector
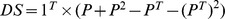


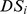
 is then the David's score for node 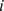 [15]. (In the case of an undirected network, the number of interactions received and given is the same so that David's score will be identically 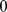.) Equivalently, a node's consensus score is given by
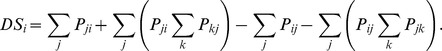
David's score has a depth of two since it requires that 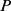 be multiplied by itself twice.

The assumptions of this algorithm are similar to the assumptions of Eigenvector Centrality. David's score favors nodes that are likely to receive interactions but unlikely to emit interactions. It also takes into consideration the partners involved in these exchanges, weighting more heavily (a) interactions received from nodes who themselves receive many interactions and (b) interactions that are sent to nodes that emit many interactions. We further discuss the differences between Eigenvector Centrality and David's score in the Text S1, Section Comparison of David's Score and Eigenvector Centrality. This process could be generalized, using paths through the network of length up to any integer 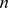, but the scores start to converge at around 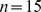, as shown in the Text S1, Section Extension of David's Score. We therefore use David's score with 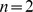 as described above.

#### Graph Laplacian

The Laplacian of a function on a network captures the amount of flow entering or leaving each node. This can be imagined as heat flowing from hot areas to cool ones. We take the Laplacian of the Shannon entropy scores 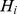 by defining the vector
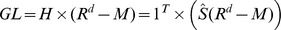
where 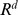 is the diagonal matrix with the 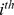 diagonal entry equal to 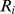, the weighted in-degree of node 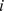. 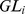 is the *Graph Laplacian Consensus* score of node 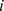. Equivalently,
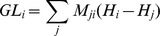
Graph Laplacian consensus has depth of two since it requires that the matrix 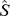 be multiplied by the matrix 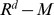.

The Laplacian should capture the amount of flow entering or leaving a node. There is a high flow from node 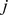 to node 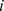 if the weight of the edge from node 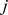 to node 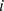 is great and the difference in 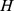 between node 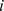 and node 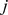 is large. A node with a high entropy of interactions received and which receives many interactions from partners whose entropy scores are low will be given a high graph Laplacian score. Therefore, although the graph Laplacian is taking into account agreement depth, the nodes it treats as more informative are those about whom there is little agreement.

### Count algorithms

Here we consider one additional algorithm, the *Borda count*, for computing consensus on networks. The Borda count is an algorithm that is traditionally used to determine the outcome of an election. Each member of a voting population ranks the candidates of the election. This is analogous to each individual in a primate group emitting signals to others in accordance with whom they perceive as more or less likely to use force successfully. The Borda count aggregates these preferences into one ranking over the candidates.

Supposing that there are 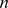 candidates, each voter gives 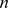 votes to his highest preference, 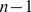 to his next highest choice, on down to one vote to his least favorite candidate. A voter can rank candidates equally and the candidates' votes in this case are the average of the numbers of votes they would have received were they not tied. A candidate's score is the sum of his votes from each voter. In the signaling case, the receiver of the most signals from a given individual will receive n “votes” and the receiver of the fewest signals from that individual will receive one “vote”. In unweighted networks, each individual “voter” divides the group into nodes with whom he does or does not interact, giving the same number of votes to the individuals in each class. Mathematically, we define a matrix 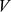 such that 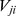 is the rank given node 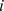 by node 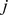, where 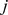 gives rank 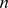 to its highest preference and rank 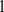 to its lowest preference, and define the vector
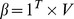


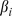
 is the *Borda consensus* scores for node 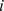.

The Borda count is more coarse-grained than the total frequency of interactions received because information about the number of interactions received is lost and only the ordinal ranking of nodes by the number of interactions received is used. It does, however, convey information about agreement among interaction partners. If we find that a node has a high score under the Borda count, this indicates that many other nodes rank the receiver highly and agree about its relative value to them. Hence like Shannon Consensus it should be intrinsically sensitive to certain kinds of bias in the interaction matrix (see Sec. Empirical Comparison for further discussion).

### Mathematical comparison

All of the algorithms we compare provide some measure of consensus in a network about the state of a given node, such that we expect they are positively correlated (these data are presented in Sec. Basics of data set). In fact, we can describe these correlations by deriving mathematical relationships between some of the algorithms. The mathematical relationships between the breadth algorithms are easiest to see. 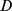, 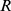 and 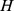 are related by the definition of 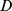 and a simple theorem about 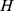:
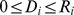


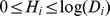
Consider the definitions of 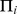 and 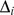:
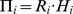


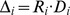
These definitions make the mathematical relationships 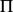 and 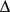 on the one hand and 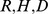 obvious.

If the network is unweighted, then we can write the following algorithms as a function of in-degree, 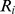:
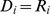


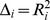


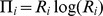


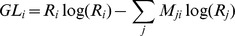


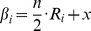
where 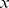 is constant across nodes and depends on the total number of edges in the network. In this case, the rankings generated by these algorithms will be the same, although the actual values will be different.

Eigenvector Centrality can be related to the redistribution probabilities and in-degree. Recall that 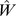 is the stochastic transition matrix where 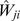 denotes the probability of walking from node 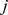 to node 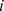 and Eigenvector Centrality is defined by the equation 
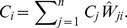
 Since 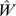 is stochastic, 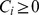 for all 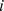 and we can choose 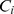 such that 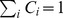. Since 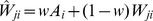, this gives 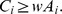 If we let 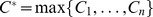, then 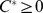 and we can show that
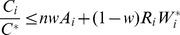
where 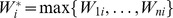. In the case that 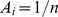 for all 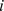, these bounds can be combined to give
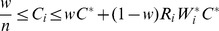
This bound gives an indication of how 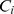 is related to the number of interactions 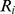 received and the redistribution weights used in the calculation of 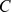. As we increase the redistribution weight 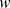, the minimum possible Eigenvector Centrality scores increases. In general, nodes that engage in more interactions and that interact with nodes with few other interaction partners will have higher Eigenvector Centrality scores.

### Empirical comparison

Much of the research on consensus aims to determine how a group comes to a single decision, such as which direction to move, who should be president, etc. [30]–[33]. In this study our aim is somewhat different. Our goal is to quantify how much consensus there is in the group (*e.g.* network) about the state of a node (is it on or off, is it capable of performing a target function, etc.). Hence the interpretation of consensus turns on the meaning of the edges in the network, represented by the data in the interaction matrix, as much as on the algorithm applied to the matrix to compute the consensus scores for the nodes. It is therefore critical that the interaction data used to construct the 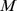 matrix be chosen carefully.

Below we provide basic details about the three test systems –a primate status communication network, a collaboration network, and a functional gene linkage network. We provide the biological interpretation of the edges in the networks and of node state, and we introduce the functional data used to empirically evaluate the algorithm's performance. We note that the mechanistic basis for consensus as an important network measurement is best understood for the primate communication network, and this fact is reflected in that section's length.

In Table 1 we provide interpretations of consensus scores for several different kinds of networks in addition to those we describe below.

### Primate communication network

We are using a primate communication network in a large captive social group of pigtailed macaques (*Macaca nemestrina*) to measure social power, operationalized as group consensus about individual 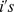 ability to win fights.

#### Background and motivation

Power dynamics are well studied in this species. Information about power structure is encoded in a network of subordination signaling interactions [14], [22]. Individuals send subordination signals (called silent bared teeth displays or *SBT*'s) to receivers they come to perceive, through a history of fighting, as likely to beat them during physical fights [34]–[36]. The signal, when emitted in peaceful settings, communicates agreement to a subordinate role in a dominance relationship [19], [22]. The signal is unidirectional (nearly always emitted by the same individual until the underlying asymmetry grows small or is reversed) [19], [34], [36].

Fighting continues after the subordination contract has been formalized through signal exchange, but at a reduced frequency [22], [35]. This provides a mechanism by which the relationship might reverse in the future. Individuals appear to roughly track the number of signals they have received, as well as the number of individuals who send them subordination signals. The data suggest that individuals use this information to determine how they are collectively perceived [14], [19]. Variation in this collective perception gives rise to the distribution of social power, where social power is defined operationally as the *consensus* opinion of group members about whether an individual can win fights [14], [21]. The data suggest that information about power is valuable to individuals because power predicts the cost individuals will pay during social interactions [17]. Individuals appear to use this information when choosing among alternative behavioral strategies [17], [23], [24].

The operational definition of social power given above is derived from a concept of social power developed in [21] that rests on the following foundational principles. Power is rooted in the use of coercion and sanctions [37]–[40]. Power is fundamentally based on perception [38], [39] about the capacity to use force successfully and is therefore subject to manipulation and error [14]. As such, power is a relational concept [37]–[39], [41] requiring repeated interactions among individuals, rather than a property of any single individual [42]. Power is normatively indeterminate [39], [43] in the sense that its exercise can result in undesirable or desirable outcomes. Power is domain specific in that it need not apply or be salient across all contexts [44]. Power must be temporally stable, and must change on a slower timescale than the underlying microscopic dynamics giving rise to it if it is to have any significant effect on behavior [14], [45]. The ability to exercise power can be undermined by transient or contingent factors, including an asymmetry in competitive motivation, third-party influences (like opportunistic coalitions), and leverage [46], [47]. Finally, although power dynamics can be a feature of pair-wise interactions as well as higher-order interactions, “social power” and the resultant power structure are network concepts [48].

Power is considered a fundamental social variable [49]. Hence a quantitative account for how a macroscopic social property like power arises from behavioral and signaling interactions would be highly informative for identifying those factors driving the consolidation of social structure from individual (low level or microscopic features) interactions.

#### Basics of data set

The data set used in our analyses to construct the signaling interaction matrix was collected over a four month period from a group of 84 pigtailed macaques (*Macaca nemestrina*) housed socially in a large compound at the Yerkes National Primate Center Field Station in Lawrenceville, Georgia (see Section Methods). The matrix contains data on pair-wise signaling interactions between socially mature individuals (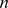 = 48). The signal is the silent-bared teeth display introduced above. The interactions are directed and the values in the matrix are weighted so that 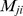 is the number of signals emitted from individual 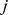 to individual 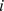.

In addition to this matrix, we have data on approximately 1000 fights (Section Methods). These data include the level of aggression interveners (third-parties to fights that become involved through their own initiation) use when intervening, the frequency with which support is solicited from third-parties, and the level of aggression received by interveners in response to their interventions. These data constitute the functional data we use to empirically validate that the algorithms measure power.

#### Note on dominance hierarchies

We note that in the animal behavior community a standard approach to ordering individuals based on dominance-related behavior is to construct a dominance hierarchy. There are many well-known algorithms for generating dominance hierarchies (*e.g.* the I&SI method developed by de Vries [27], [28]) but they are ill-suited as measures of consensus because many of these algorithms order pairs of individuals so that the order minimizes the number of intransitivities in a matrix containing information about dominance interactions (*e.g.* wins, subordination signals, etc.). This is problematic because the degree of transitivity is a critical factor influencing how much consensus there is in the group about an individual's state and algorithms that explicitly or implicitly take the degree of transitivity into account are desirable. In addition, the rank order generated by these kinds of dominance hierarchy algorithms is strictly the outcome of pairwise assessments, rather then the outcome of a whole group or population-level assessment. Finally, the rank order produced by these algorithms is typically ordinal. At least with respect to the study of power, a scalar measure is desirable so that the the impact of variance in the distribution of power on individual strategy and decision-making can be studied. (We note, however, that in social systems with a normal distribution of fighting abilities and perfect learning, there should be no intransitivities in dominance relationships and in such cases the distribution of power will best be described by a uniform distribution that can be captured by an ordinal rank order [50].)

#### Note on consensus about low status

In principle it is possible to assess consensus about the *inability* to win fights (low status) using the same algorithms we have presented in this paper. However, assessments about low status would require a different data set. The reason for this is that the subordination signal used in our primate analyses is unidirectional and always emitted by the opponent perceiving itself as weaker. Consequently signal emission *only tells us* that the sender perceives the receiver to have high status. *Quantifying consensus about low status requires a signal that indicates the sender perceives the receiver as weaker*. Although these signals, called *formal dominance signals*, are from an information theoretic standpoint not as reliable indicators of status, some species do use them (issues reviewed in [19]). The absence of signal exchange in contrast can indicate many things, including that the (1) individual withholding the signal perceives itself as stronger, (2) individual withholding the signal perceives itself as equivalent, and (3) that the individual withholding the signal has no opinion.

### Physics collaboration network

We are using a collaboration network to measure reputation, defined operationally as group consensus about whether to work with a given scientist.

#### Background and motivation

In the scientific community, a collaboration can be interpreted as an indication of complementary skill sets and ideas. Encoded in a collaboration network is a collective perception of an individual's scientific quality. One might predict that an individual with many collaborators is widely perceived to be a good scientist and is consequently more likely to receive funding to support his or her research.

#### Basics of data set

A well-studied science collaboration network is the condensed matter physicist network compiled by Newman [15]. There are 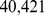 condensed matter physicists represented in the collaboration network, as reported in [15]. The interactions are unweighted and undirected so that 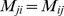 is 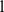 if scientists 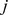 and 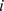 collaborated and 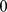 otherwise. We additionally have independent data on grants awarded to 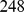 of those scientists. We evaluate whether measures of consensus applied to this physics collaboration network gives a reputation index that predicts NSF grant-related success (see Section Methods for data set details and definitions).

### Functional gene linkage network

We are using a network of functional linkages between genes to measure gene importance, defined operationally as group consensus about whether to be functionally linked with a given gene (this “decision” could be made in either developmental or evolutionary time).

#### Background and motivation

In recent years, data on the interactions between genes has become readily available, particularly in the model system baker's yeast, *Saccharomyces cerevisiae* [6]–[10], [51]–[56]. Data include the phenotypic effects of genes, the physical properties of the proteins that are encoded by genes, and information about gene function [6]–[10], [51]–[56]. If a gene interacts with many other genes, removal might have long-ranging effects. Some studies have found that a gene's position in functional linkage networks is a good predictor of its function and of the likely effects of a mutation [7], [9], [10], [53].

We evaluate whether measures of consensus applied to a functional gene linkage network produce an index of “importance” scores that predicts the effects of gene knockout on organismal growth and viability as described below and in Section Methods.

#### Basics of data set

To quantify consensus about gene importance we use a previously described gene linkage network that describes functional linkages between the genes in the yeast genome [51], [56]. Functional linkages between genes are associations that “represent functional constraints satisfied by the cell during the course of the experiments” [56]. To establish this network, functional genomic data were combined from multiple types of experiments and databases to determine whether two genes are involved in a similar function [51], [56] (see Section Methods). The interactions are unweighted and undirected so that 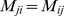 is 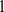 if there is evidence for a functional linkage between genes 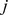 and 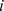 and 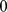 otherwise.

We additionally use information about the phenotypic effects of genes in the yeast genome (see Section Methods). Two phenotypic effects reflect a gene's overall importance. One measure is the viability of organisms with a mutant version of the gene and a second measure is the competitive fitness of organisms with a mutant version of the gene. We call genes whose mutations lead to inviable organisms essential and genes whose mutations lead to viable organisms non-essential. These data are available for 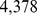 of the 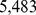 genes in the linkage network. We expect to find that essential genes and those whose mutations lead to decreased fitness will be more central or more highly connected in the functional linkage network and will therefore receive higher scores from our algorithms.

### Algorithm outputs

Each algorithm produces as output a vector of scores for nodes in the network. In Table S1 in Text S1 we present the correlations between these outputs for each network . The distribution of consensus scores for each of the networks, according to each of the algorithms considered in this paper, is presented in Figures S2, S3 and S4. Within each data set, most algorithms suggest roughly similar distributions. In the case of the signaling network, these distributions look heavy-tailed, which is consistent with the distributions of functional data. Additionally, for the signaling network, two of the algorithms– Shannon Consensus and Weighted Simple Consensus– produce distributions that are not significantly different than normal after log transform, indicating they are consistent with the log-normal distribution.

### Prediction of functional data

#### Criteria and issues

There are two main criteria we use for evaluating the algorithms, (a) how well the indices produced by the algorithms predict differences in function or behavior, and (b) how sensitive the algorithms are to source biases in the interaction matrix due to systematic error or attempts to manipulate the output by strategically changing the input. An algorithm that is insensitive to source biases would, for example, accord a high score to a node simply because its total weighted in-degree is high. We say such an algorithm is insensitive when it cannot “see” whether the input to the target node is coming from many nodes in the network, a small subset of nodes, or only a single node. For example, in the case of power, an algorithm that accords a high score to an individual who receives many signals but receives them all from a single individual, would be considered insensitive to source bias [14]. Although we tried to choose algorithms that by virtue of their mathematical properties are sensitive source biases, this is not always obvious, so we test for sensitivity to bias as part of our analyses.

To evaluate the algorithms' performance on the two criteria introduced above, we must take into account two issues.

#### Prediction heterogeneity

**Figure 1:**
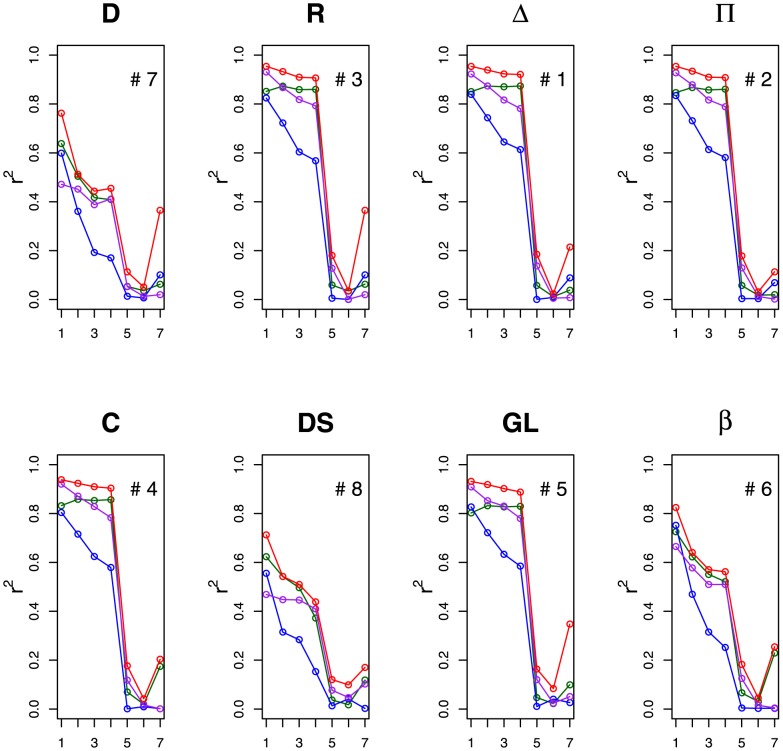
This figure shows for the primate communication network the fit of each algorithm to the functional data. The x-axis indicates which subset of nodes are being considered– 1 is the top quartile, 2 is the top half, 3 is the top three quartiles, 4 is all nodes, 5 is the bottom three quartiles, 6 is the bottom half, and 7 is the bottom quartile– where the quartiles may vary from algorithm to algorithm (see Section heterogeneity). The 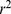 values for the three dependent variables are distinguished by color: support solicited (green), aggression used (blue), intervention cost (purple). The multivariate 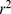 values are shown in red. The number in the each plot indicates the rank of each algorithm with respect to its performance predicting the functional data. As expected, we find that the consensus scores for the top-ranked nodes are most predictive of the functional data (see, Section Prediction heterogeneity).

Distributions of scores that range over several orders of magnitude can result in *prediction heterogeneity*. For example, in a heavy-tailed distribution many nodes will have similar scores, whereas those sitting towards the tail of the distribution will be more distinct. Any index with this kind of distribution should be highly predictive for nodes in the tail and less predictive for nodes in the bulk of the distribution, as these nodes are more or less “equivalent”. To control for prediction heterogeneity without biasing the analyses *a priori* against other types of distributions, we rank, according to the output of each algorithm, nodes by their score and divide them into four subgroups. The subgroups are slightly different for each algorithm because the rank orders differ across algorithms. We regress the dependent variables on the scores in top quartile, the top two quartiles, the top three quartiles, all nodes, the bottom three quartiles, the bottom two quartiles, and the bottom quartiles (Figures 1, S5 and S6).

#### Timescales

Recall that the subordination signals the monkeys exchange are unidirectional. This property makes the signals highly reliable indicators that the sender perceives the receiver as capable of using force. This also allows individuals to formalize their dominance relationships. A consequence (and advantage) of this formalization is that the dominance relationships change on a slower timescale than the outcome of fights [22], effectively producing a separation of timescales. This separation of timescales means we can disentangle the question of how much consensus there is in the group that an individual is powerful from the study of how individuals come to perceive one another as powerful. In addition, because the power distribution is stationary over the study period ([22]), we can study its impact on individual strategy without worrying about feedback. Similarly, the collaboration network and the data on NSF funding success come from two nearly non-overlapping time periods. (The collaborations occurred between January, 1995 and March, 2005 and the grants we use as a measure of success were awarded between September, 2008 and September, 2012, with one grant starting in September, 2004.) In the gene network, there is separation of timescales between the the formation of functional linkage network, which occurs on an evolutionary timescale, and the phenotypic effects of each gene, which occur on an ontogenetic timescale. In evolutionary time, the phenotypic effects of a gene surely feedback to effect which other genes its interacts with, but given an organism with a certain functional linkage network it is the topology of the interaction network that influences how important each gene is.

### Primate communication network: Predicting effects of social power

Our predictor variables are the social power indices produced by the consensus algorithms. Dependent variables include: support solicited – requests for support received by a third-party to a fight from fight participants (should be positively correlated with power); intervention cost – operationalized as the intensity of aggression received by an intervener in response to its interventions into fights among group members (should be negatively correlated with); and intensity of aggression used by an intervener during its intervention (should be negatively correlated) (these variables are defined and the data collection methods are described in Section Methods and in [14]). These dependent variables are corrected for underlying variation in tendency to fight (see Section Methods).

All algorithms are significantly correlated with the dependent variables (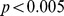, Table 4). The best predictor of the dependent variables is Weighted Simple Consensus, followed closely by Shannon Consensus, and Eigenvector Centrality. The worst predictors are David's Score, Borda Count, and Simple Consensus. The most highly predictive algorithms have very similar 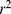 values, so it is hard to differentiate between them based on their predictive power alone. However, as we discuss below, these algorithms vary in their sensitivity to source biases and in their computational and cognitive complexity.

**Table 4:** Tables of the predictive value of the scores produced by each algorithm for all nodes on the three data sets.

	Support Solicited		Intensity of Aggression Used		Intervention Cost		Multivariate	
	*r* ^2^	p-value	*r* ^2^	p-value	*r* ^2^	p-value	*r* ^2^	p-value
*D*	0.41	<0.001	0.17	.003	0.41	<0.001	0.45	<0.001
Δ	0.87	<0.001	0.61	<0.001	0.78	<0.001	0.92	<0.001
Π	0.86	<0.001	0.58	<0.001	0.79	<0.001	0.91	<0.001
*C*	0.86	<0.001	0.58	<0.001	0.78	<0.001	0.90	<0.001
*DS*	0.37	<0.001	0.15	0.005	0.41	<0.001	0.44	<0.001
*GL*	0.83	<0.001	0.58	<0.001	0.78	<0.001	0.89	<0.001
*BC*	0.52	<0.001	0.25	<0.001	0.51	<0.001	0.56	<0.001

(a). Here we report the *r* ^2^ values and p-values for a linear regression of the functional data against the algorithm scores from the primate communication network. Basics of the data set and functional data are described in Section basics of data set.		
	Total Grants Awarded	
	*r* ^2^	p-value
*D*	0.004	0.33
Δ	0.000	0.83
Π	0.002	0.45
*C*	0.025	0.01
*GL*	0.004	0.33
*BC*	0.004	0.33

(b). Here we report the *r* ^2^ values and p-values for a linear regression of the functional data against the algorithm scores from the physicist collaboration network. Basics of the data set and functional data are provided in Section basics of data set.				
	Essentiality		Competitive Fitness	
	t-statistic	p-value	*r* ^2^	p-value
*D*	15.64	<0.001	0.04	<0.001
Δ	10.46	<0.001	0.02	<0.001
Π	14.38	<0.001	0.04	<0.001
*C*	15.66	<0.001	0.04	<0.001
*GL*	7.20	<0.001	0.01	<0.001
*BC*	15.64	<0.001	0.04	<0.001
**(c). Here we report the t-statistics and p-values for comparing scores of essential and non-essential genes and the *r*^2^ values and p-values for a linear regression of competitive fitness against the algorithm scores from the functional linkage network. In using a t-test to compare the mean scores for essential and non-essential genes, the p-values are for a one-sided test with the alternative hypothesis that essential genes have greater scores. Basics of the data set and functional data are given in Section basics of data set.**				

In this social system, there are a few individuals in the tail of the power distribution who are disproportionately powerful [14], [17]. This is borne out in our data, as the correlation between the algorithm scores and the dependent variables is substantially higher for the top quartiles than the bottom quartiles (Figure 1).

### Collaboration network: Predicting effects of reputation

Our predictor variables are the reputation indices produced by the consensus algorithms. The dependent variable is total amount of grant money awarded to a PI or CoPI by the National Science Foundation (see Section Methods). Of all the algorithms we consider, only Eigenvector Centrality is significantly correlated with this external variable (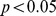, Table 4). Two reasons, one mathematical and one sociological, appear to account for this result.

First, Eigenvector Centrality can distinguish between nodes that have identical local neighborhoods. In-degree can only take integer values and there is presumably an upper bound on the number of possible collaborators given time and other constraints. In this network, the highest in-degree observed is 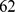 so that there are 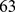 possible values, 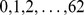, a node's in-degree can take. As Eigenvector Centrality can take any value between 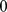 and 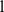, it can give different scores to nodes with the same in-degree. In other words, Eigenvector Centrality uses global information to differentiate between nodes that are locally identical. This effect is not as noticeable in the subordination signaling network because there are only 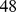 individuals in the signaling network and therefore less degeneracy in the in-degree distribution.

Second, it is perhaps not surprising that for this kind of network Eigenvector Centrality is more predictive of the dependent variable than the breadth algorithms – although physicists involved in the process of awarding grants to others are expected to recuse themselves when confronted with an application from one of their own collaborators, they may be more likely to award grants to collaborators of their collaborators. Therefore, having many collaborators may not be that helpful in receiving grant money, but scientists whose collaborators have many collaborators may have an advantage.

### Functional gene linkage network: Predicting effects of importance

Our predictor variables are the importance indices produced by the consensus algorithms. The dependent variables are the viability and competitive fitness of organisms with mutated versions of the gene. For each of our algorithms, the importance scores for essential genes are significantly higher than the importance scores for non-essential genes (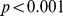, Table 4). Similarly, for each algorithm, the importance scores are significantly negatively correlated with the competitive fitness variable (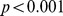,Table 4). The most predictive algorithms are, in order, Eigenvector Centrality 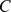, Simple Consensus 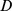, the Borda count, and Shannon Consensus 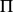. In differentiating between essential and non-essential genes, Eigenvector Centrality is marginally better than the other algorithms. In predicting competitive fitness, the four most predictive algorithms perform equally well.

With both external variables, the test statistics are noticeably smaller for the Graph Laplacian than for the other algorithms. As we showed above, on unweighted networks,
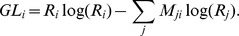
On both the collaboration and linkage networks, nodes with high in-degree tend to interact with many other highly connected nodes. For both networks, we find high correlations between in-degree, 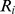, and the sum of the in-degrees of a node's neighbors, 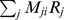 (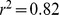, 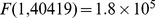, 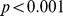 for the collaboration network and 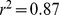, 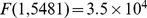, 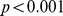 for the linkage network). Nodes that have many interactions with other highly connected nodes receive low Graph Laplacian scores, a counterintuitive result that suggests the Graph Laplacian is not a robust measure of consensus. We summarize the predictive performance of the algorithms on the three data sets in Table 5.

**Table 5:** Summary of data sets and the most highly predictive algorithms, in order of their performance predicting the functional data.

Network	Functional Data	Most Predictive Algorithms
Primate communication network	support solicited, aggression used, intervention cost	Δ, Π, *C*, *GL*
Collaboration network	grants awarded	*C*
Gene functional linkage network	viability of mutants, competitive fitness	*C*, *D*, *BC*, Π

Only the algorithms that significantly predict the functional data are included. Note that in many cases the differences in performance across the algorithms are small. In addition, the *r* ^2^ values are small for the functional gene network and the physicist collaboration network and large for the primate communication network. This difference is probably due to the fact that the subordination signals are direct measures of power in the primate network, whereas the edges in the other networks are either indirect/proxy measures of reputation and importance or are only one of many contributors to the variance.

### Sensitivity of the algorithms to source biases

An important question in evaluating the performance of a consensus algorithm is how sensitive the algorithm is to deficiencies in the data in the interaction matrix. Aspects of this question have been addressed in previous work. Ghoshal et al. [57] showed that in scale-free networks of sufficient size, if all edges in the network are shuffled but the in-degrees maintained, the ranking of the nodes according to eigenvector centrality is not severely perturbed. This type of shuffle allows the researcher to simulate the effects of missing or noisy data in the interaction matrix on an algorithm's output. We are particularly interested in the effects on the algorithm's output of nodes systematically making errors in their assessments of the states of other nodes or nodes attempting to manipulate social structure by “loading the deck” or inflating the consensus scores of nodes by, for example, manipulating the weighted degree distribution. (One way to manipulate the weighted degree distribution is to inflate a node's weighted in-degree by sending many signals.) Capturing this kind of “deficiency,” which we call *source bias* requires a different kind of shuffle.

First, we measure in our interaction matrices the correlation between a node's Shannon entropy (as defined in Sec. Shannon consensus) and the total frequency of interactions it receives (weighted in-degree or in-degree) (see Table S1 in Text S1). If entropy and in-degree were poorly correlated, we could independently evaluate the effects of receiving many interactions from receiving interactions from many individuals. However, this is not the case on the data sets we consider. We break the correlation by systemically shuffling the data in the matrices such that we create matrices with strong source biases but conserve the total number of interactions (*e.g.* signals) received. We now have two matrices –the original, unshuffled matrix, 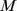 and the shuffled matrix, 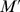. We then compute consensus scores for the nodes using the unshuffled and shuffled matrices and assess how much the rank order changes under the shuffle.

More specifically, for a given pair of interaction partners in the network, say nodes 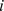 and 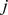, we construct a matrix in which the target node, 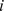 receives all of its interactions from partner node, 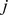. If the original network is directed, we hold constant the out-edges of 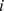 in addition to holding constant 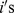 weighted in-degree. If the original network is undirected, we maintain the symmetry. The subordination signaling network is small enough so that we can perform this shuffle for every pair of partners. However, the collaboration network and the functional linkage networks are too large to exhaust every pair of partners, so we choose 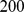 of the nodes that are also represented in the functional data sets. Partner nodes are chosen at random from the target node's neighbors.

An algorithm is said to be sensitive to source bias if the rank order of the shuffled matrix, 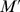 differs from the rank order of the original matrix, 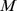. Large changes in the rank order indicate that the test algorithm tends to give higher scores to nodes that interact with many neighbors than to nodes that interact strongly with just one other node and is an indication that the algorithm is sensitive to source biases.

**Figure 2:**
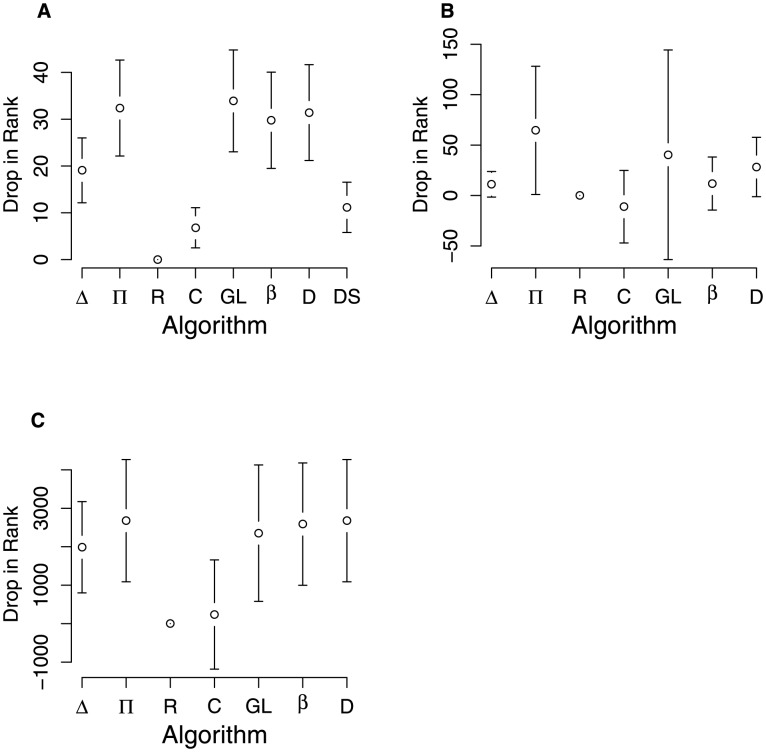
This figure shows show the sensitivity of each algorithm to source bias in the interaction matrix. For each algorithm, we report the drop in rank induced when a node receives all of its edges from one of its neighbors. The point shows the mean correlation and the bars show plus or minus one standard deviation. The algorithms are ordered from left to right by their predictive power for the primate communication network. In the case of the primate communication network, we exhaust all possible pairs and in the case of the collaboration and functional linkage networks, we choose 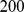 at random. **A**. Primate communication network. **B**. Physicist collaboration network. **C**. Functional linkage network of genes.

We find that Shannon Consensus 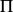, and the Graph Laplacian, 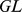, tend to be quite sensitive to source biases (Figure 2). This is expected, as 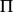 and 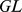 depend on the entropy of the receiving distribution, which is by design 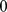 in the shuffled matrices.

By definition, in-degree, 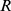, is maximally insensitive to source bias as we hold it constant in our shuffle. Eigenvector Centrality, 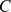, is also fairly insensitive, but the explanation why is initially counter-intuitive. As can be seen in Figure 2, Eigenvector Centrality appears to be particularly insensitive to the shuffle for the subordination signaling network, as the rank order for the shuffled and unshuffled matrices for that network is very similar. The reason for this is that in the subordination signaling network individuals who receive many signals receive some of these signals from partners who themselves receive many signals. In addition individuals who receive many signals send very few signals. Hence there is information about breadth encoded in the second and third order connections (and so forth) in the network. Even if we shuffle the matrix so that all of an individual's signals come from a single other node, as long as we hold constant the out-edges of 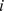, the in-edges to 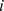 are likely to be from an individual who itself receives relatively many signals. Eigenvector Centrality, by emphasizing paths through the network, takes these second and third order connections into account. It is consequently likely to get the rank order right, even after we reduce the diversity or breadth in the first order or direct connections, as long as the second and third order connections in the shuffled matrices encode information about the first order connections in the unshuffled matrices. See Text S1, Section Sensitivity of eigenvector centrality on transitive networks for more discussion of the relationship between transitivity and sensitivity to source bias.

This suggests that measures of consensus that emphasize depth– paths through the networks –also implicitly measure consensus breadth *when there is some degree of either assortativity or transitivity in the network, and work well because of these features*. In the absence of transitivity, or when transitivity is very low, depth measures like Eigenvector Centrality, should not perform well as measures of consensus, unless, as in the case of the Graph Laplacian, they explicitly incorporate Shannon information. In the Text S1 we provide details on an additional analysis we performed to evaluate algorithm sensitivity to source bias.

## Discussion

The algorithms we investigate take as an input a matrix of physical or social interactions. These interactions encode “perceptions” or “opinions” about a target node's state or abilities. The algorithms produce as an output scores that quantify agreement or consensus in the population about this state or ability. We find that the best performing algorithms (in terms of prediction and robustness) are those that capture the breadth of agreement among a node's population of partners (see Table 5). Capturing the breadth of agreement requires quantifying the diversity of the target node's population of partners and weighting this by the number of interactions. Our analyses suggest that algorithms with these properties are robust to source biases in the interaction matrix arising from efforts to manipulate output or when nodes make systematic errors in assessing the state of a target node.

We find that in a primate status communication network, the most predictive algorithm is Weighted Simple Consensus, which is also one of the more computationally minimal algorithms we consider. In contrast to the class of algorithms that make use of Shannon entropy, Weighted Simple Consensus is not particularly sensitive to source bias. This suggests a tradeoff between computational complexity and sensitivity to source bias.

In the physicist collaboration network indirect measures of an author's (the target node) state are important, as decisions about the target node's reputation can be based on the reputation of its associates. Depth-based algorithms like Eigenvector Centrality capture these indirect effects. One caveat is that these algorithms only work as measures of consensus when networks are characterized by an elevated degree of transitivity or assortativity.

We conclude that in general the uniformity based algorithms are preferable but that Eigenvector Centrality is suitable if the network is transitive or assortative and if there is a mechanistic reason to believe that it is important to take second and third order connections into account. We discuss these issues in greater detail below.

### Strengths and weaknesses of the algorithms

#### Breadth algorithms

The strength of the breadth algorithms is that they clearly capture consensus. Shannon Consensus captures consensus in a strong sense by quantifying uniformity in the signaling distribution through entropy. This is reflected in the fact that Shannon Consensus is sensitive to source bias, i.e. changing an node's signaling distribution is disruptive to the scores output by Shannon Consensus. Simple Consensus and Weighted Simple Consensus are also predictive and have the advantage of being easier to calculate and so cognitively more parsimonious. This is an important consideration if a goal is to understand how nodes in the network are estimating how much consensus there is among other nodes about their states, as would be the case, for example, when individuals are making strategic decisions based on estimates of their power. We discuss the issue of computational complexity in greater detail in Sec. Cognitive and computational complexity and in the Text S1, Section Computational Complexity.

An obvious potential limitation of these algorithms is that they consider only interactions of reach one (immediate neighbors) in the signaling network. Furthermore, all partners are weighted equally regardless of how many interactions they have with their neighbors. This could be a weakness if nodes who themselves receive many interactions are either more important sources of information or better informed, or if nodes must rely on indirect information to make decisions, as might be the case for the collaboration network (See Section Collaboration network: Predicting effects of reputation).

#### Depth algorithms

The strength of the depth algorithms is that they quantify agreement depth, such that the interactions of nodes who themselves receive interactions from their neighbors are weighted more heavily than the interactions of nodes receiving few interactions from their neighbors. We suggested in Section Collaboration network: Predicting effects of reputation that long-range ties are important in predicting physicists' grant-receiving success, as individuals involved in granting awards may be discouraged from favoring their direct collaborators, but not the collaborators of their collaborators. In networks where nodes not only can but are encouraged to use information about long-range ties, the depth algorithms may perform better than the breadth algorithms. However, we emphasize that this is likely only to be the case if these networks contain many transitive relationships or are assortative.

Given that the depth algorithms use more global information about the network topology, they can help to differentiate between nodes whose positions in the network are locally similar. As we found with the physicist collaboration network, the ability of Eigenvector Centrality to distinguish between nodes to which the breadth algorithms gave identical scores made it more predictive of the functional data. This could happen on any network that has many nodes and where there is a constraint on the possible number of in-edges, leading to multiple nodes sharing the same in-degree.

Eigenvector Centrality can inappropriately assign high scores to nodes simply because they receive many interactions from a small group of partners This will not be a problem if the networks are characterized by transitive relationships. However it is worth noting that for such networks, the global structure used in calculating Eigenvector Centrality may not be more informative than the local structure used in the breadth algorithms.

The Graph Laplacian can be quite sensitive to source bias. However, as we found with the collaboration and functional linkage networks, if highly connected nodes are connected to other highly connected nodes, the graph Laplacian produces a consensus index that is not predictive of the functional data. This suggests that the Graph Laplacian is not a good measure of consensus.

#### Count algorithms

The advantages of the Borda Count are that it is a relatively simple measure to compute, and that it overcomes many of the inconsistencies that arise in consensus-based approaches to assigning value to individuals based on scoring. Arrow's impossibility theorem is one formulation of such inconsistencies (although it is not clear that this is a problem when the aim is to generate a consensus index rather than an optimal rank order) [58], [59]. A disadvantage is that it is highly-coarse grained.

Whether the Borda Count contains information about agreement among signalers depends on our definition of agreement. Nodes receiving the same number of signals from many nodes will not necessarily receive a high Borda count. However, nodes that receive high Borda Counts are those whom all nodes signal to the most frequently. This expresses agreement about relative rankings if not about absolute strength.

That the Borda Count does not well predict the functional data in the case of the subordination signaling network suggests it is not capturing consensus. Additionally, on unweighted networks, the Borda count is linearly related to in-degree. The Borda count is more complex to calculate and does not provide any additional information in the unweighted case, and hence we conclude it is not a good measure of consensus.

### Cognitive and computational complexity

The results reported in this paper and elsewhere([17], see also [60]–[62]) suggest that at least in social networks nodes may be making strategic decisions about social interactions using knowledge of how they are perceived by the group. For example, the individuals in the primate study group appear to estimates of their relative power to make decisions about whether to intervene in conflicts [17]. This requires that they have some knowledge of moments or properties of the distribution of power (*e.g.* approximate variance). An important question is how individuals extract this information [22], [63]. More generally, what do animals know about social structure and collective dynamics, how precise are their estimates, and what heuristics might they use to make calculations [64]?

It would be useful, for example, to be able to quantify the algorithmic complexity of each algorithm so that we could rank calculations by some measure of computational difficulty (see also [65]). Ideally, we would also like to know how sensitive each algorithm is to the input data. (*e.g.* is the exact number of signals received by individual 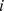 critical, or will a rough estimate do?) for the output distribution of power to be a useful predictor of out of sample data. Addressing this robustness question would help to determine how much room there is to relax the mathematical requirements of a given algorithm, and find a heuristic simple enough for this study species.

Ranking the algorithms by their algorithmic complexity is a long way off, if achievable at all. As is illustrated in Figure S1, we can only crudely rank the algorithms given what we know about the minimum number of steps each requires in order to estimate critical quantities from an empirical perspective – the absolute power of individual 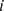, the relative power of 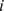 (*e.g.* where it falls in a power distribution of a given type), and the moments of the power distribution. In most circumstances it seems unlikely that we, or the animals, would be interested in an isolated individual's score. This is because it is not her power value that is important, but rather where an estimated value falls in a distribution of power scores. Yet calculation of absolute and relative power require different computational approaches and a preliminary assessment suggests that the difficulty of these steps varies across algorithms. We discuss these issues in greater detail in Text S1, Section Computational complexity.

In addition to approaching the problem of complexity mathematically, we can approach it empirically by asking how sensitive the algorithms are to imperfect information in the input matrices. For example, perhaps the individuals in our system cannot discriminate based on identity and can only remember classes of individual (*e.g.* male or female, or matriline x or y, etc.), 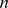 signals or 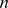 signalers, or an interaction history of length 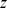. By coarse-graining the input data, it is in principle possible to test how sensitive the algorithms are to this kind of imperfect information resulting from various cognitive or spatial constraints. Aspects of this question have been addressed in previous work, as discussed in Section Sensitivity of the algorithms to source biases. However, many questions remain open for future work.

### Broader implications for the study of social structure

If node function in many different systems is collectively encoded in interaction networks and this information is decodable by quantifying the agreement in network connectivity patterns, this would suggest that consensus formation is at the core of sociality. Consider the primate society used as a model system in this paper.

Power in our primate study group is a critical social variable. However power is not a simple variable. The distribution of power does not map directly onto a distribution of body sizes or even a distribution of fighting abilities. Rather it consolidates as multiple interacting individuals learn about fighting abilities and signal about this to reduce social uncertainty [14], [19], [21], [22], [63]. When the statistics used to operationalize an aggregate social property, like power structure, are more than simple counts over strategies, and when the inputs are not simply individual traits but network data, we need to worry explicitly about the mappings between behavioral strategies and decision-making at the microscopic level and social organization [65]–whether we are working with the social organization of primates or of cells forming a tissue. A central question becomes, How do strategies get collectively combined by multiple components to produce macroscopic social properties? How much degeneracy characterizes this mapping? Once we can describe the developmental dynamics giving rise to an aggregate social property, we will be in a position to study how the social processes producing power and other kinds of social structure have evolved in a wide range of systems.

## Methods

### Primate communication network

The data set, collected by J.C. Flack, is from a large, captive, breeding group of pigtailed macaques that was housed at the Yerkes National Primate Research Center in Lawrenceville, Georgia.

#### Ethics statement

All data on the macaque group were collected in compliance with the ethical standards set by the Emory University animal care and welfare committee and IACUC approval (proposal 216-97) was obtained to conduct the study. As this was an observational study, the only change to the daily routine of the animals that was required to collect the data was that the animals had to be confined to their outdoor housing during each observation period. Water, monkey chow (remaining from morning feeding), enrichment (*e.g.* toys, climbing structures, etc.) and substantial space were available continuously throughout all observation periods. On very hot or rainy days, observations were terminated and the monkeys were given access to their indoor housing. As part of standard Yerkes management protocol, the animals were routinely subject to medical examination and care.

#### Study system

Macaque societies are characterized by social learning at the individual level, social structures that arise from nonlinear processes and feedback to influence individual behavior, frequent non-kin interactions and multiplayer conflict interactions, the cost and benefits of which can be quantified at the individual level (*e.g.* [14], [17], [19], [23], [24], [66], [67]). These properties make the macaque genus and its representative species excellent choices for drawing inferences about critical processes in social evolution as well as for developing new modeling approaches that are intended to apply more broadly.

The study group had a demographic structure approximating wild populations. Subadult males were regularly removed by the Yerkes animal care staff to mimic emigration occurring in wild populations (no subadults were removed during the study). The group contained 84 individuals, including 4 adult males, 25 adult females, and 19 subadults (totaling 48 socially-mature individuals used in the analyses). All individuals, except 8 (4 males, 4 females), were either natal to the group or had been in the group since formation. The group was housed in an indoor-outdoor facility, the outdoor compound of which was 125×65 ft.

Pigtailed macaques are indigenous to southeast Asia and live in multi-male, multi-female societies characterized by female matrilines and male group transfer upon onset of puberty [66]. Pigtailed macaques breed all year. Females develop swellings when in oestrus.

#### Data collection protocol

During observations all individuals were confined to the outdoor portion of the compound and were visible to the observer. The approximately 156 hours of observations occurred for up to eight hours daily between 11AM and 830PM over a twenty-week period from June until October 1998 and were evenly distributed over these hours. Provisioning occurred before observations, and once during observations. The data were collected over a four-month period during which the group was stable (defined as no reversals in status signaling interactions resulting in a change to an individual's power score).

Conflict and power (subordination signal) data were collected using an all-occurrence sampling procedure in which the compound was repeatedly scanned from left to right for onset of conflict or the occurrence of silent-bared teeth displays (used to measure power, see below). When a conflict erupted, the entire conflict event was followed, and data were collected on start time, end time, and the identity of individuals involved and their behavior (see below for operational definitions). Although conflicts in this study group can involve many individuals, participation is typically serial, making it possible to follow the sequence of interactions. A nearly complete time-series of conflict events is available for each observation period.

#### Operational definitions

Conflict/Fight: includes any interaction in which one individual threatens or aggresses a second individual. A conflict was considered terminated if no aggression or withdrawal responses (fleeing, crouching, screaming, running or backing away, submission signals) occurred for two minutes from the last such event. A conflict can involve multiple pairs if pair-wise conflicts result in aggressive interventions by third parties or redirections by at least one conflict participant. In addition to aggressors, a conflict can include individuals who show no aggression (*e.g.* recipients or third-parties who either only approach the conflict or show affiliative/submissive behavior upon approaching). Because conflicts can involve more than two individuals, two or more individuals can participate in the same conflict but not interact directly.

Agonistic Dyad: includes interaction between pairs within a conflict. At least one individual in the pair must direct aggression towards the other. Individual 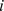 is said to be the winner if its opponent, individual 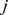 exhibits withdrawal-related behavior in response to the behavior of 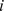 and this withdrawal-related behavior is not superseded by aggressive behavior towards 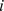 at any point during the conflict. Individual 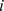 is said to be the loser if it is the one to show withdrawal-related behavior. The interaction is said to result in a draw if both 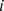 and 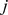 direct aggression towards each other and this aggression is not superseded by withdrawal-related behavior, or if one individual directs aggression towards the other and the other shows no response.

Subordination Signal: the subordination signal in the pigtailed macaque communication repertoire is the peacefully-emitted variant of the silent bared-teeth display. Bared-teeth (BT) displays are marked by a retraction of the lips and mouth corners such that the teeth are partially bared. In pigtailed macaques, the SBT occurs in two contexts: peaceful and agonistic [19]. Signals in both contexts are highly unidirectional. The agonistic SBT encodes submission. The peaceful variant signals agreement to a primitive social contract in which the signaler accepts the subordinate role [19].

Dependent variables used to test empirical validity of algorithms are described in detail in [14]. Briefly, they include frequency of requests for support received by an individual from fight participants (should be positively correlated with power), intervention cost incurred by an individual operationalized as the intensity of aggression received by an intervener in response to its interventions into fights among group members (should be negatively correlated), and intensity of aggression used by an intervener during its intervention (should be negatively correlated). These dependent variables are corrected for underlying variation in tendency to fight by calculating an expected score based on the group average (for a given variable) and subtracting this from the observed score. Details are in [14].

### Physics collaboration network

The physicist collaboration network was collected by Mark Newman, as described in [38], and is available at http://www-personal.umich.edu/~mejn/netdata/. The data were initially collected from the Los Alamos e-Print Archive, now the arXiv at http://arxiv.org. Since initial publication in 2001, the network has been updated with collaborations from the arXiv through 2005. 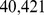 scientists are represented in the network and the collaborations occurred between January, 1995 and March, 2005.

The National Science Foundation makes the data about awarded grants publicly available at http://www.nsf.gov/awards/about.jsp. For each scientist in the collaboration network, we searched this database for any grant concerning condensed matter physics on which the scientist was one of the investigators. If the scientist was awarded more than one grant, we summed the total amount of grants awarded him or her. Grant data was available for 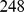 of the 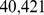 scientists in the collaboration network. The grants were awarded between September, 2008 and September, 2012, with one grant starting in September, 2004.

### Functional gene linkage network

The functional linkage network was constructed by Lee et al., as described in [56] and [51], and is available at http://www.yeastnet.org/. Functional linkages between genes are associations that “represent functional constraints satisfied by the cell during the course of the experiments” [56]. Evidence of a functional linkage between two genes was provided by mRNA coexpression levels, the results of protein interaction experiments, phylogenetic profiles, and the co-occurrence of the two genes in a scientific paper [51], [56]. Lee et al. combined these data to calculate the log-likelihood that two genes are involved in a similar function. In our analyses, we say an edge is present if its log-likelihood score is greater than 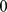 and is absent otherwise. The resulting network has 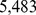 nodes.

The *Saccharomyces* Genome Database maintains information about the phenotypic effects of genes in the yeast genome at www.yeastgenome.org. Two phenotypic effects reflect a gene's overall importance. One measure is the viability of organisms with a mutant version of the gene and a second measure is the competitive fitness of organisms with a mutant version of the gene. The viability measure is binary: a mutation to a gene can lead to either a viable or an inviable organism. An inviable organism is one that is unable to grow under standard growth conditions for *S. cerevisiae*, defined as glucose-containing rich medium (YPD) at 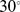
C. A gene's competitive fitness is given by the relative growth rate of an organism with a mutated version of the gene compared to one with the normal genotype. Greater competitive fitness is indicated by a relative growth rate of greater than 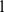. These experiments can be performed in various media: we only used those performed in minimal medium to standardize our comparisons. More information is available at http://www.yeastgenome.org/help/function-help/phenotypes. These phenotype data are available for 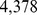 of the 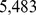 genes in the linkage network.

## Supporting Information

Figure S1 **Flow chart for the calculation of the various algorithms.** The color indicates our intuitions about the complexity of each algorithm, with darker grays corresponding to more complex calculations.
(PDF)

Figure S2 **The distribution of the scores resulting from each algorithm as applied to the subordination signaling network.**
(PDF)

Figure S3 **The distribution of the logarithms of the scores resulting from each algorithm as applied to the physicist collaboration network.**
(PDF)

Figure S4 **The distribution of the scores resulting from each algorithm as applied to the yeast functional linkage network.**
(PDF)

Figure S5 **This figure shows for the physicist collaboration network the fit of each algorithm to the functional data.** The x-axis indicates which subset of nodes are being considered– 1 is the top quartile, 2 is the top half, 3 is the top three quartiles, 4 is all nodes, 5 is the bottom three quartiles, 6 is the bottom half, and 7 is the bottom quartile– where the quartiles may vary from algorithm to algorithm (see Section Prediction heterogeneity). The number in the each plot indicates the rank of each algorithm with respect to its performance predicting the functional data.
(PDF)

Figure S6 **This figure shows for the functional linkage network of genes the fit of each algorithm to the functional data.** The x-axis indicates which subset of nodes are being considered– 1 is the top quartile, 2 is the top half, 3 is the top three quartiles, 4 is all nodes, 5 is the bottom three quartiles, 6 is the bottom half, and 7 is the bottom quartile– where the quartiles may vary from algorithm to algorithm (see Section Prediction heterogeneity). The number in the each plot indicates the rank of each algorithm with respect to its performance predicting the functional data.
(PDF)

Figure S7 **The worst case error of the naive entropy estimator as a function of the naive estimator.** Each data point represents the naive entropy estimate and worst case error of one individual's receiving distribution. Figure was created by Simon DeDeo, 2011.
(PDF)

Figure S8
**This figure shows how predictive value of eigenvector centrality changes as a function of the redistribution parameter.** **A**. shows how the 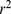 value from a multivariate regression of eigenvector centrality on the subordination signaling network against the external data depends on the redistribution probability. **B**. shows how the 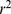 value from a regression of eigenvector centrality on the physicist collaboration network against the external data depends on the redistribution probability. **C**. shows how the 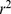 value from a regression of eigenvector centrality on the functional linkage network against competitive fitness depends on the redistribution probability. In the subordination signaling network and the physicist collaboration network, eigenvector centrality is most predictive when the redistribution probability is about 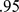. In the gene interaction network, eigenvector centrality is most predictive when the redistribution probability is about 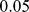.(EPS)

Figure S9
**This figure shows how predictive value and skewness of eigenvector centrality scores are related.** **A**. shows how the 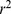 value from a multivariate regression of eigenvector centrality on the primate communication network against the external data depends on the skewness of the scores. **B**. shows how the 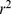 value from a regression of eigenvector centrality on the physicist collaboration network against the external data depends on the skewness of the scores. **C**. shows how the 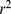 value from a regression of eigenvector centrality on the functional linkage network against competitive fitness depends on the skewness of the scores. In the primate communication network, the redistribution weight that minimizes skewness is 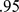. In the collaboration network, the weight that minimizes skewness is 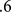. In the gene interaction network, the redistribution weight that minimizes skewness 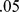. These points are filled in.(PDF)

Figure S10
**This figure shows the effects of varying the length of the random walk,** 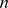 **, on the power scores computed using David's Score.** The 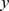 axis shows David's score for each individual, normalized so that the highest score is 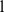 and the lowest score is 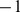. Each line corresponds to the generalization of David's score for different 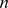, as specified in the Figure legend. The plot shows that at about 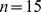 the rank orders begin to converge.(PDF)

Figure S11 **This figure shows show the sensitivity of each algorithm to source bias in a shuffled primate communication matrix.** For each algorithm, we report the drop in rank induced when a node receives all of its edges from one of its neighbors. The point shows the mean correlation and the bars show plus or minus one standard deviation. The algorithms are ordered from left to right by their predictive power for the primate communication network. **A**. Primate communication network. **B**. We remove the transitivity in the primate communication network by constructing a random network where each node has the same in-degree and out-degree as in the primate communication network. On this network, eigenvector centrality is no longer significantly less sensitive than the other algorithms.
(PDF)

Figure S12 **Each plot shows the (normalized) entropy scores and (normalized) algorithm scores for the four algorithms that do not correlate well with entropy on four artificial data sets constructed to show this lack of correlation.** The solid lines are the entropy scores and the dashed lines are the algorithms.
(PDF)

Text S1 **The Supporting Information contains the following sections: 1. Sample entropy issues 2. Empirical evaluation of redistribution weights for Eigenvector Centrality 3. Extension of David's Score 4. Comparison of David's Score and Eigenvector Centrality 5. Sensitivity to source bias on transitive networks 6. Mathematical intuition for insensitivity to source biases 7. Computational complexity.**
(PDF)
