# Supplementary figures and images for: A Family of Algorithms for Computing Consensus about Node State from Network Data
Source: PLoS Comput Biol. 2013 Jul 18;9(7):e1003109. doi: 10.1371/journal.pcbi.1003109 (PMC3715438; doi:10.1371/journal.pcbi.1003109)

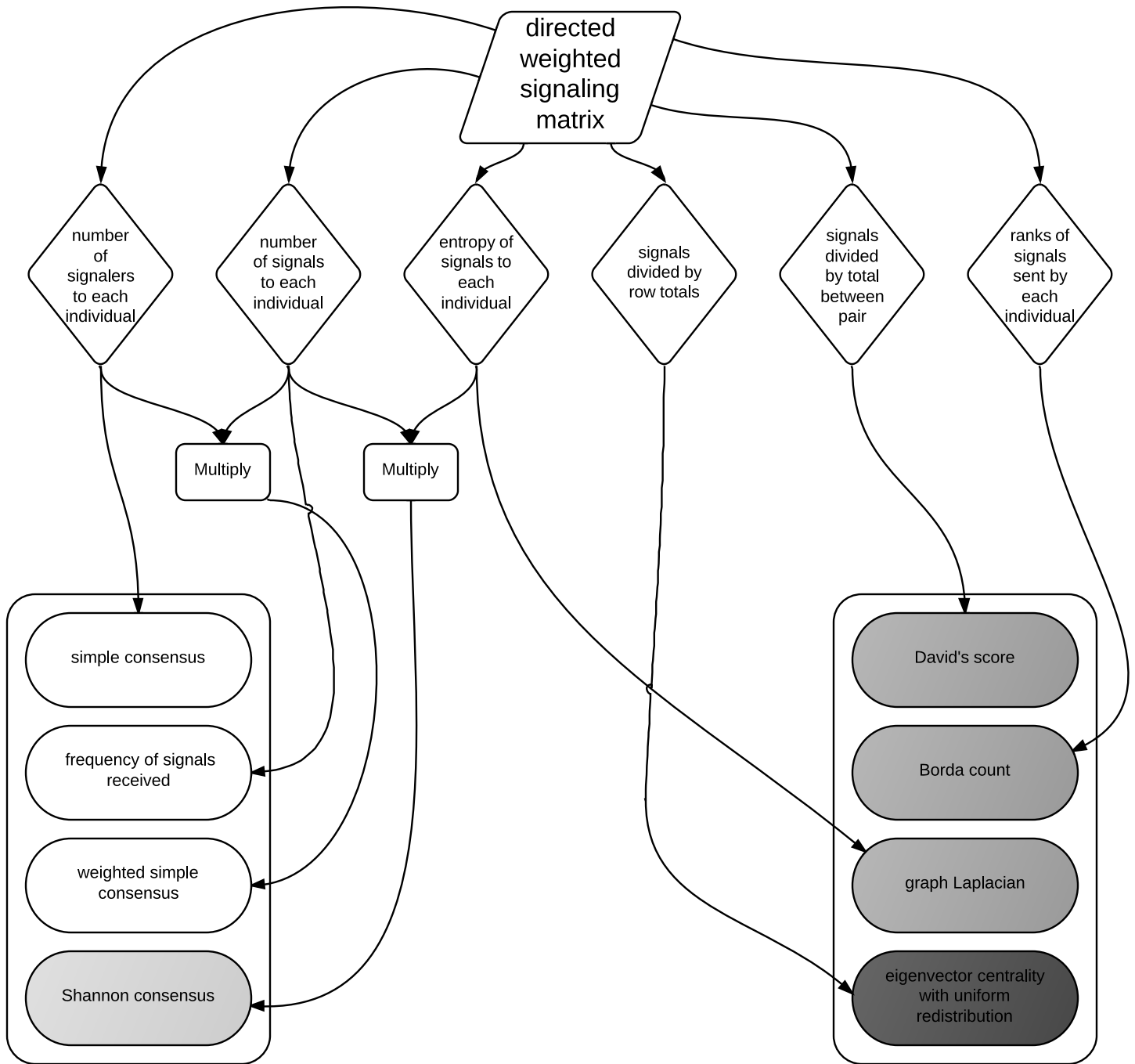

Supplement: Figure S1 — Flow chart for the calculation of the various algorithms. The color indicates our intuitions about the complexity of each algorithm, with darker grays corresponding to more complex calculations. (PDF) [file pcbi.1003109.s001.pdf]

**D**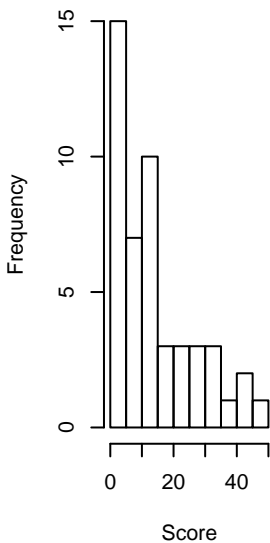**R**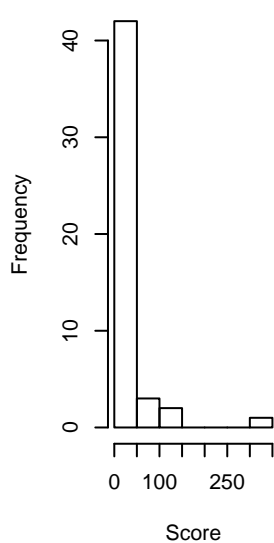 $\Delta$ 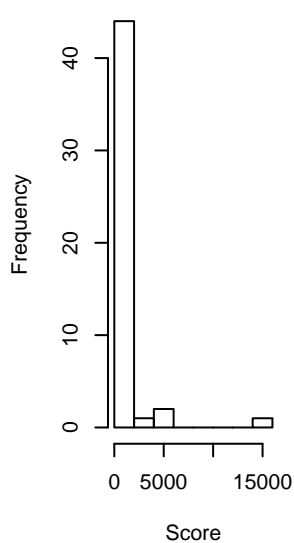 $\Pi$ 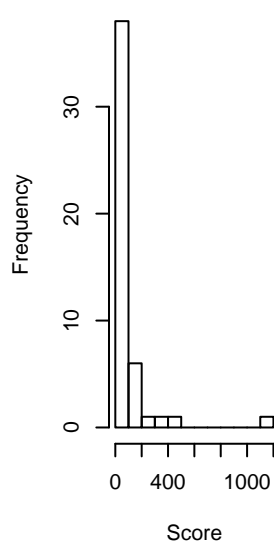**C**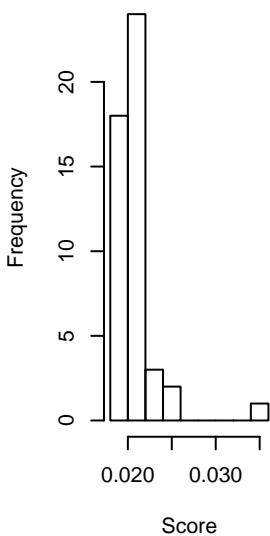**DS**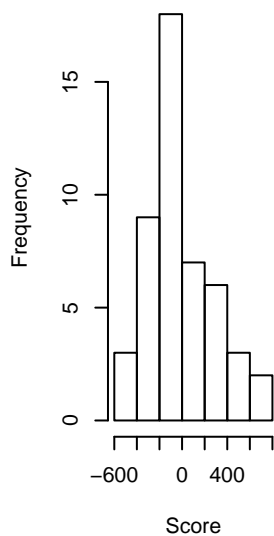**GL**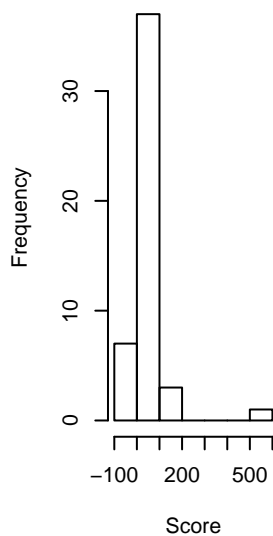 $\beta$ 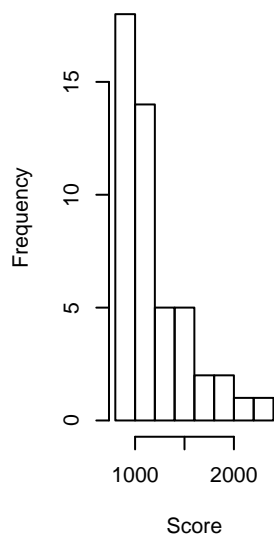

Supplement: Figure S2 — The distribution of the scores resulting from each algorithm as applied to the subordination signaling network. (PDF) [file pcbi.1003109.s002.pdf]

**D**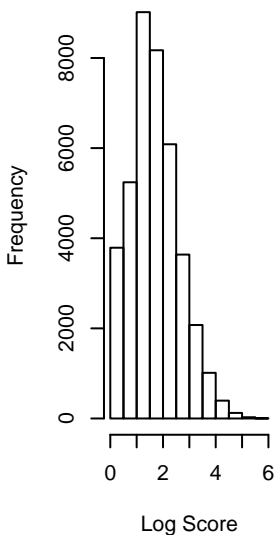**R**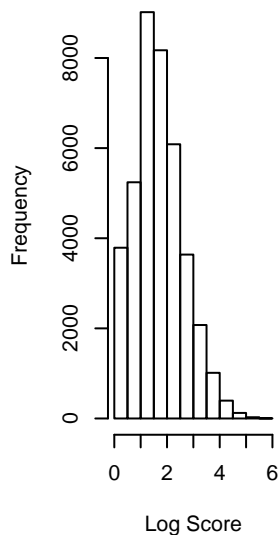 **$\Delta$** 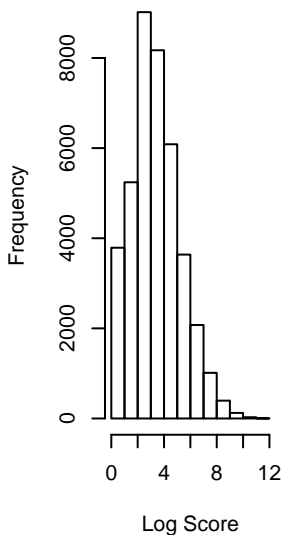 **$\Pi$** 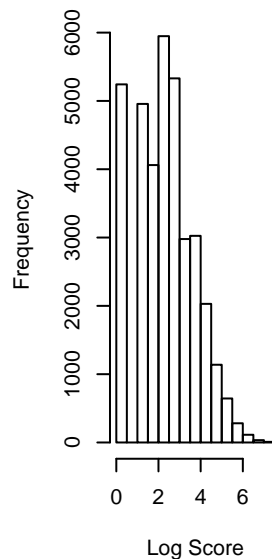**C**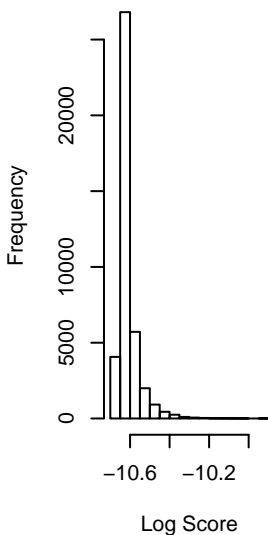**GL**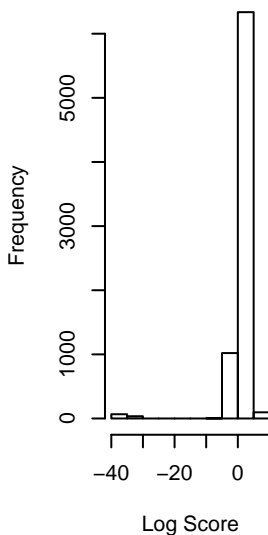 **$\beta$** 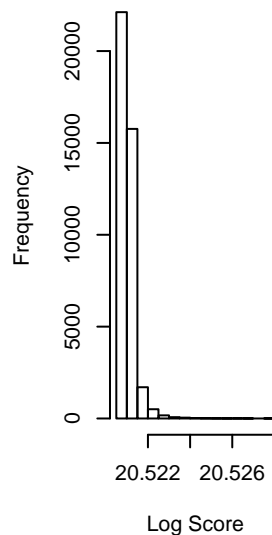

Supplement: Figure S3 — The distribution of the logarithms of the scores resulting from each algorithm as applied to the physicist collaboration network. (PDF) [file pcbi.1003109.s003.pdf]

**D**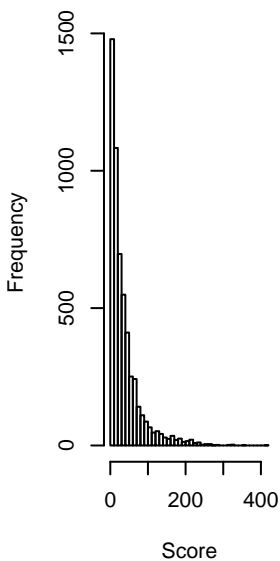**R**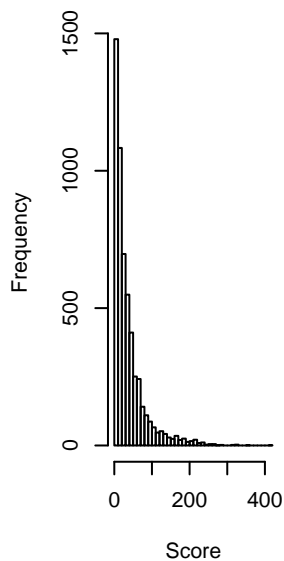 $\Delta$ 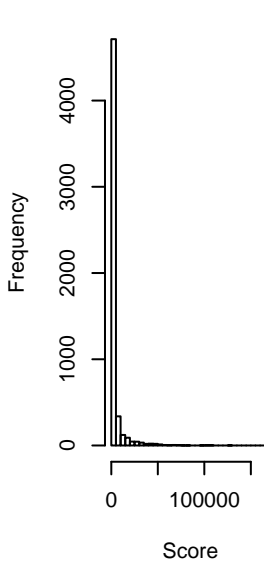 $\Pi$ 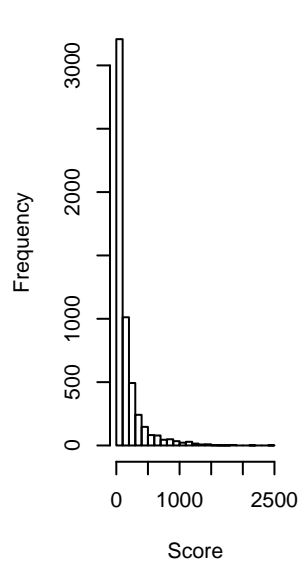**C**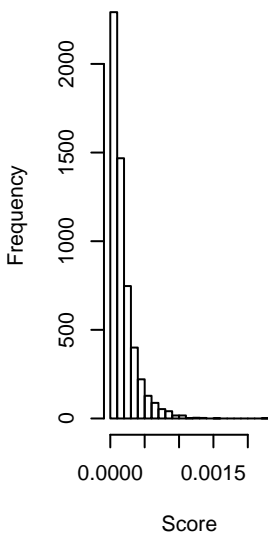**GL**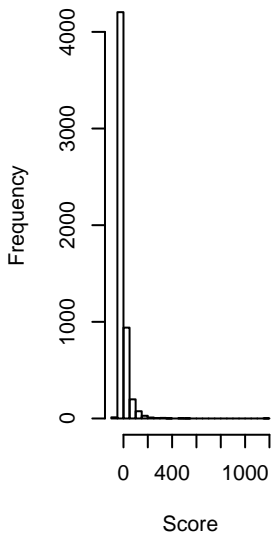 $\beta$ 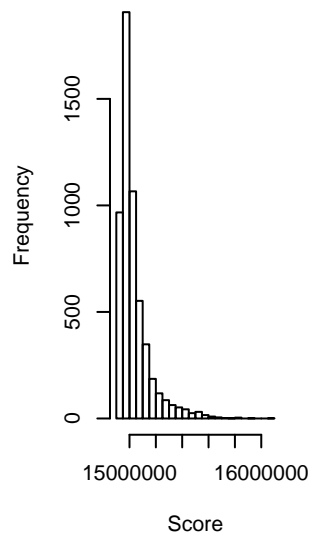

Supplement: Figure S4 — The distribution of the scores resulting from each algorithm as applied to the yeast functional linkage network. (PDF) [file pcbi.1003109.s004.pdf]

**D**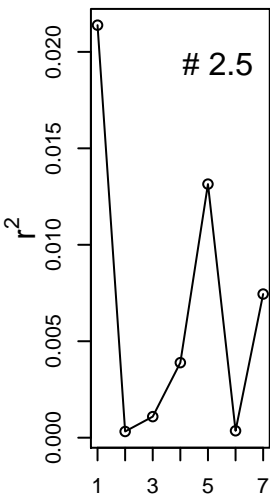**R**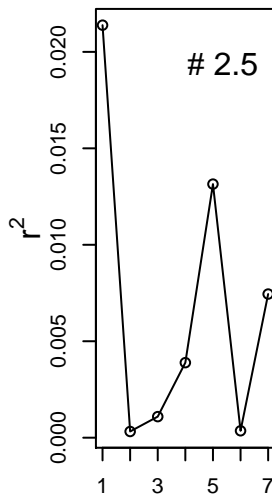 $\Delta$ 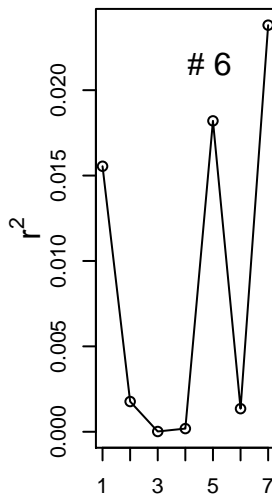 $\Pi$ 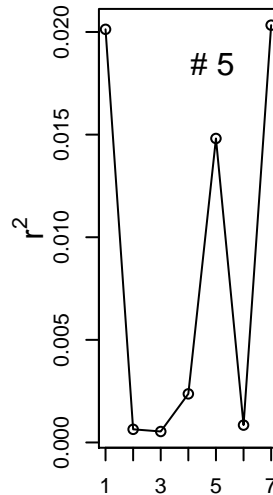**C**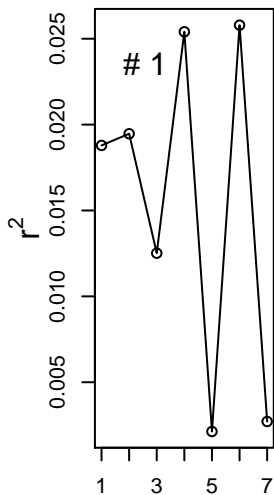**GL**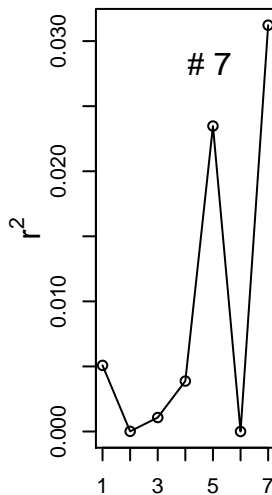 $\beta$ 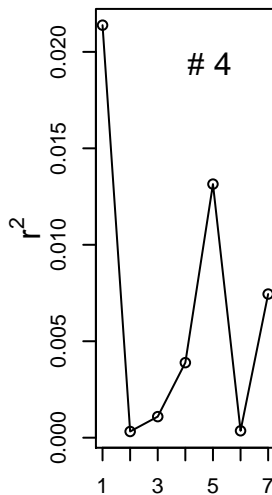

Supplement: Figure S5 — This figure shows for the physicist collaboration network the fit of each algorithm to the functional data. The x-axis indicates which subset of nodes are being considered– 1 is the top quartile, 2 is the top half, 3 is the top three quartiles, 4 is all nodes, 5 is the bottom three quartiles, 6 is the bottom half, and 7 is the bottom quartile– where the quartiles may vary from algorithm to algorithm (see Section Prediction heterogeneity). The number in the each plot indicates the rank of each algorithm with respect to its performance predicting the functional data. (PDF) [file pcbi.1003109.s005.pdf]

**D**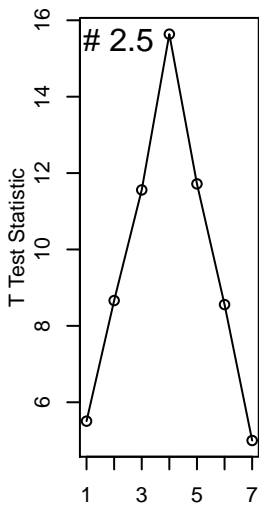**R**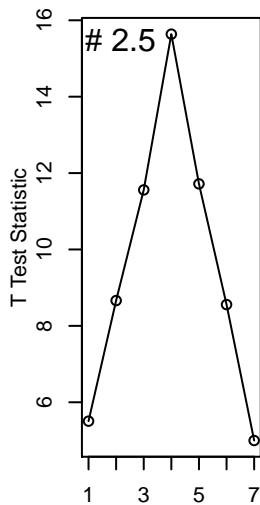 $\Delta$ 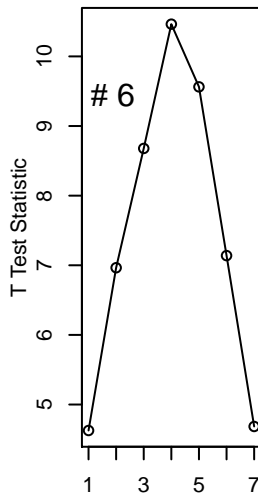 $\Pi$ 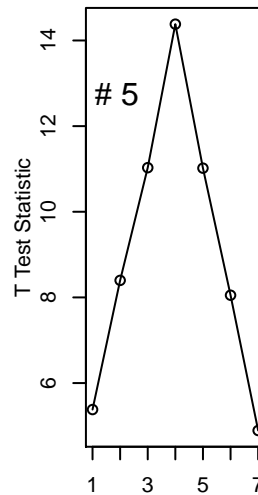**C**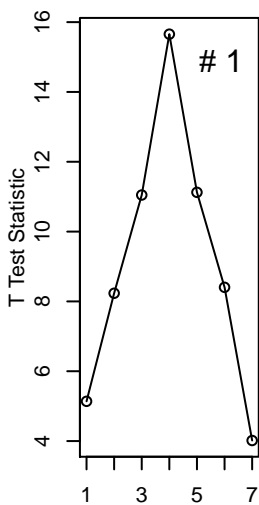**GL**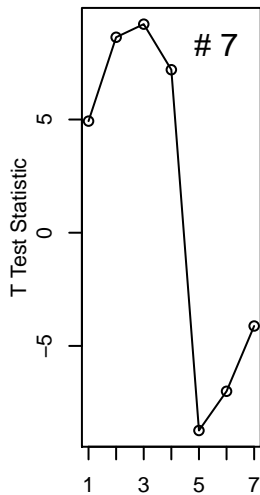 $\beta$ 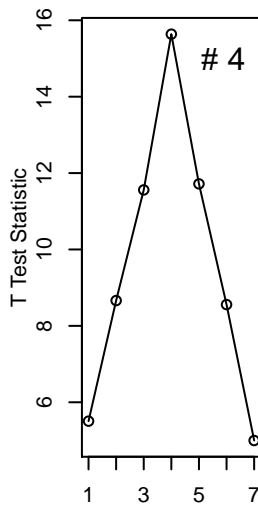

Supplement: Figure S6 — This figure shows for the functional linkage network of genes the fit of each algorithm to the functional data. The x-axis indicates which subset of nodes are being considered– 1 is the top quartile, 2 is the top half, 3 is the top three quartiles, 4 is all nodes, 5 is the bottom three quartiles, 6 is the bottom half, and 7 is the bottom quartile– where the quartiles may vary from algorithm to algorithm (see Section Prediction heterogeneity). The number in the each plot indicates the rank of each algorithm with respect to its performance predicting the functional data. (PDF) [file pcbi.1003109.s006.pdf]

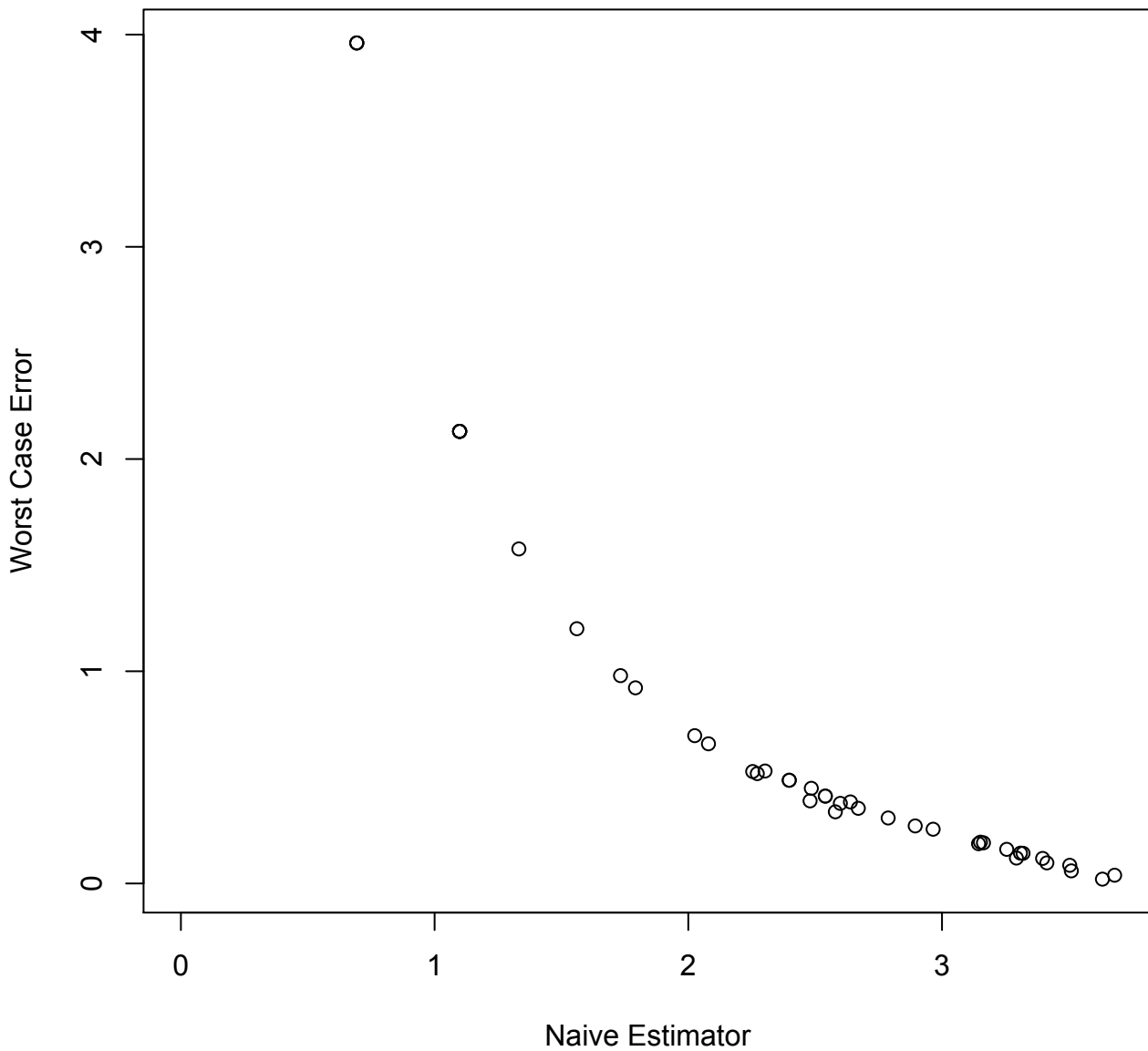

Supplement: Figure S7 — The worst case error of the naive entropy estimator as a function of the naive estimator. Each data point represents the naive entropy estimate and worst case error of one individual's receiving distribution. Figure was created by Simon DeDeo, 2011. (PDF) [file pcbi.1003109.s007.pdf]

**A**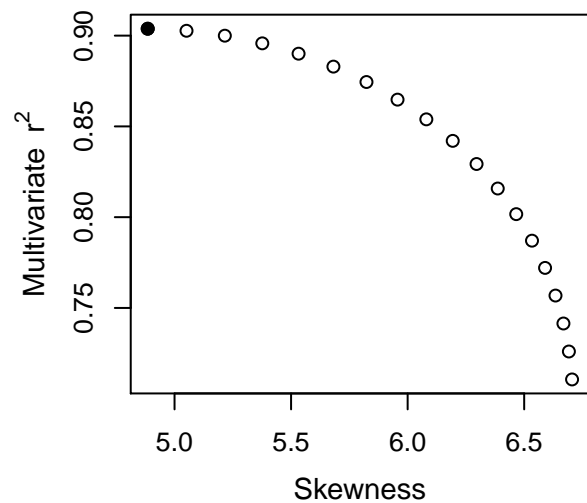**B**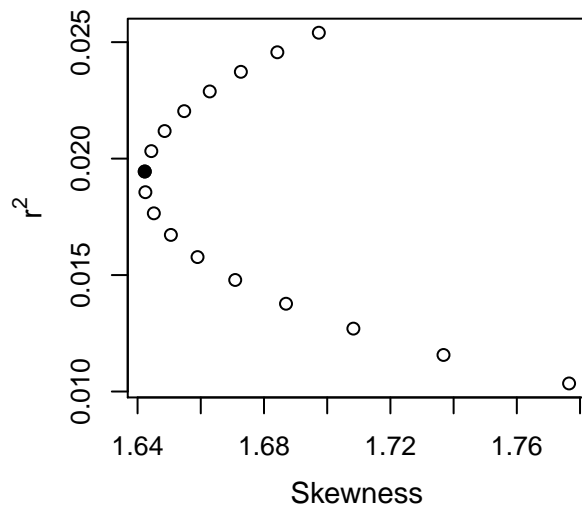**C**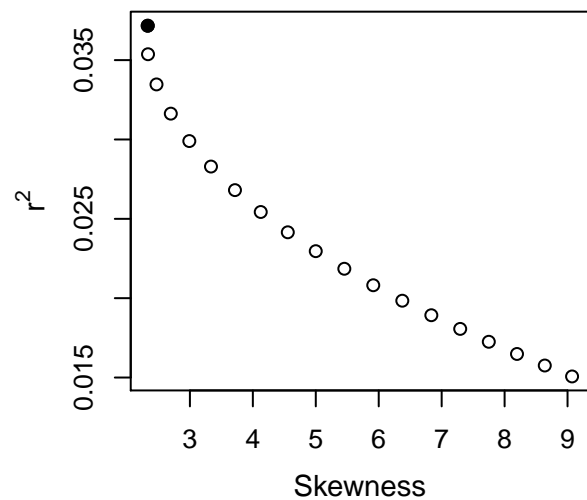

Supplement: Figure S9 — This figure shows how predictive value and skewness of eigenvector centrality scores are related. A. shows how the value from a multivariate regression of eigenvector centrality on the primate communication network against the external data depends on the skewness of the scores. B. shows how the value from a regression of eigenvector centrality on the physicist collaboration network against the external data depends on the skewness of the scores. C. shows how the value from a regression of eigenvector centrality on the functional linkage network against competitive fitness depends on the skewness of the scores. In the primate communication network, the redistribution weight that minimizes skewness is . In the collaboration network, the weight that minimizes skewness is . In the gene interaction network, the redistribution weight that minimizes skewness . These points are filled in. (PDF) [file pcbi.1003109.s009.pdf]

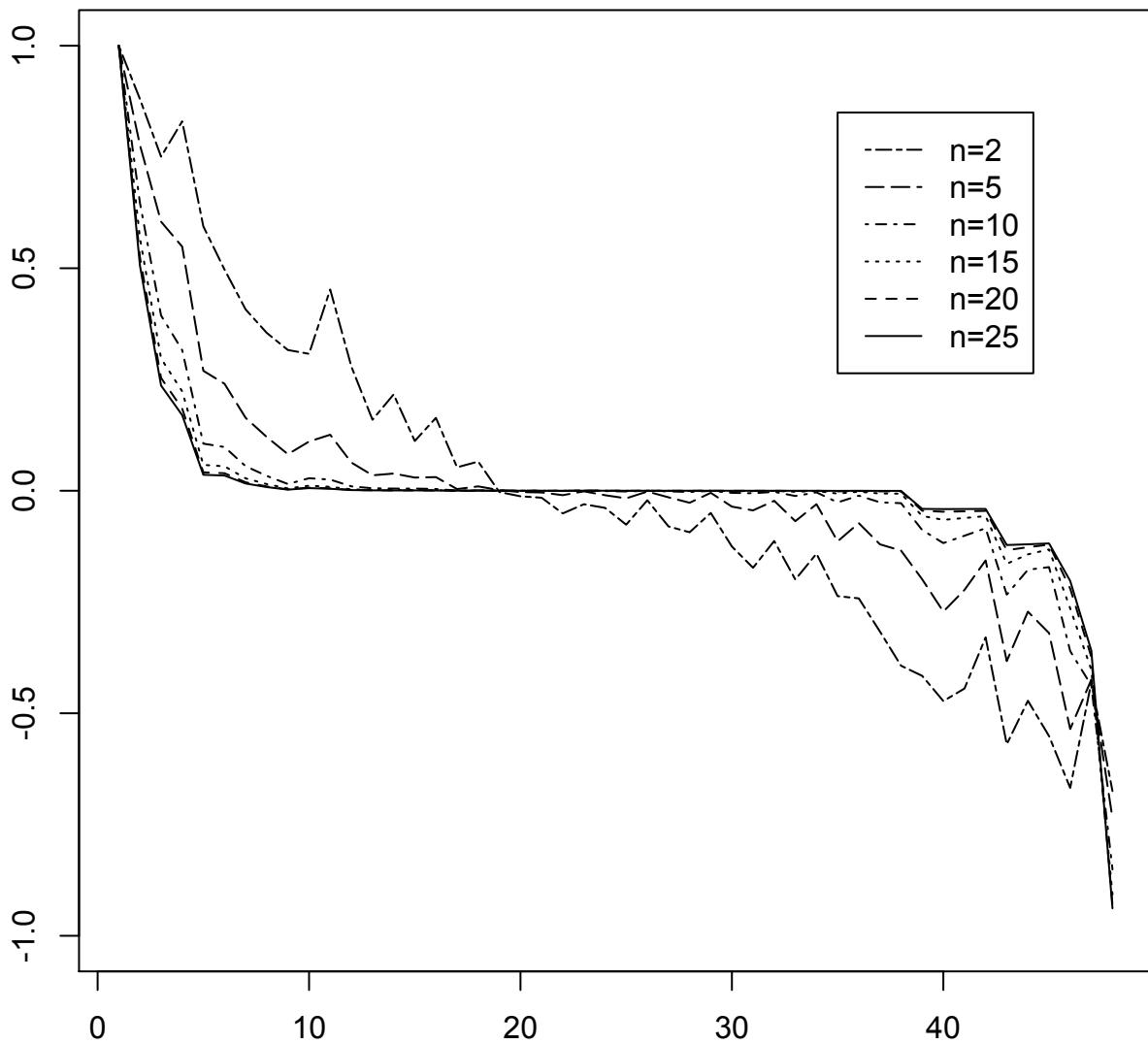

Supplement: Figure S10 — This figure shows the effects of varying the length of the random walk, , on the power scores computed using David's Score. The axis shows David's score for each individual, normalized so that the highest score is and the lowest score is . Each line corresponds to the generalization of David's score for different , as specified in the Figure legend. The plot shows that at about the rank orders begin to converge. (PDF) [file pcbi.1003109.s010.pdf]

**A**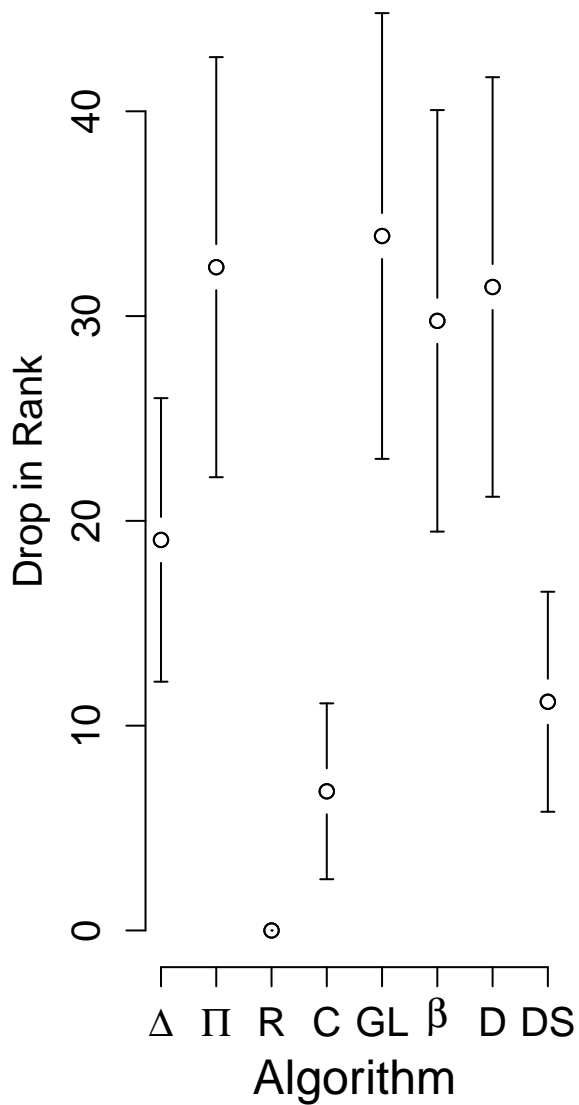**B**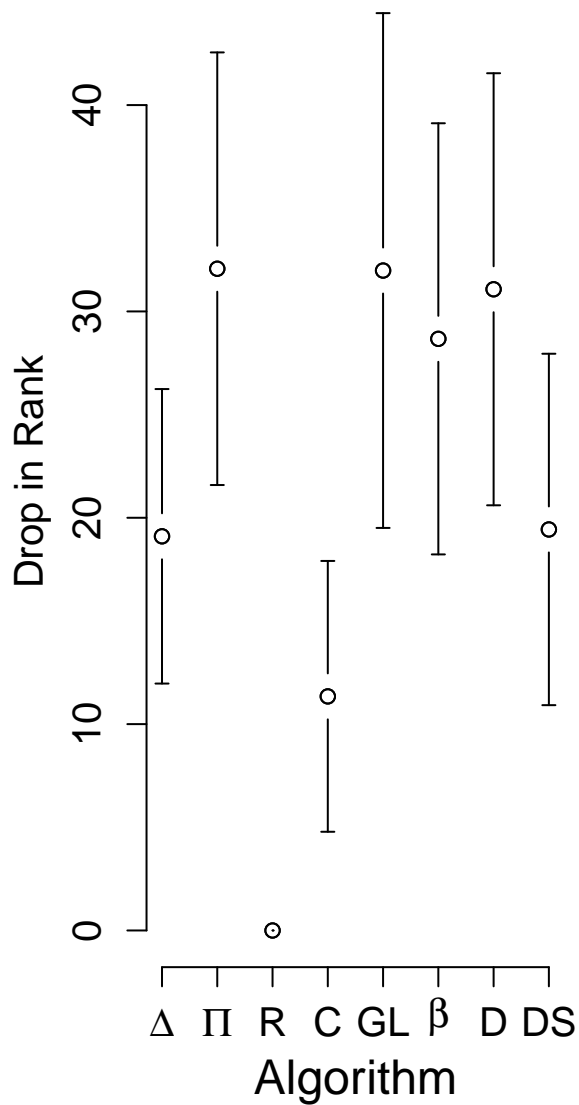

Supplement: Figure S11 — This figure shows show the sensitivity of each algorithm to source bias in a shuffled primate communication matrix. For each algorithm, we report the drop in rank induced when a node receives all of its edges from one of its neighbors. The point shows the mean correlation and the bars show plus or minus one standard deviation. The algorithms are ordered from left to right by their predictive power for the primate communication network. A. Primate communication network. B. We remove the transitivity in the primate communication network by constructing a random network where each node has the same in-degree and out-degree as in the primate communication network. On this network, eigenvector centrality is no longer significantly less sensitive than the other algorithms. (PDF) [file pcbi.1003109.s011.pdf]

**C**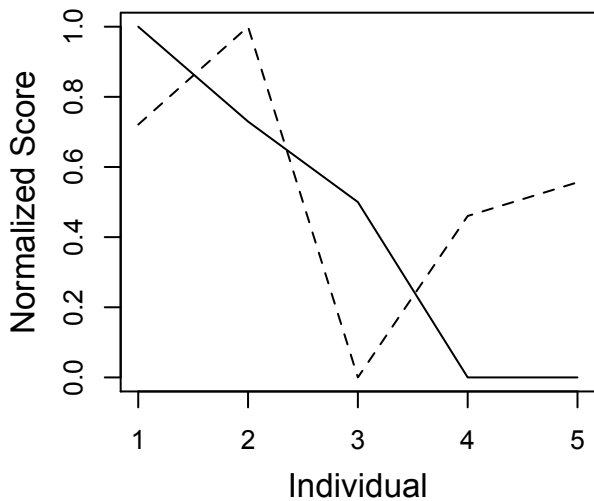**DS**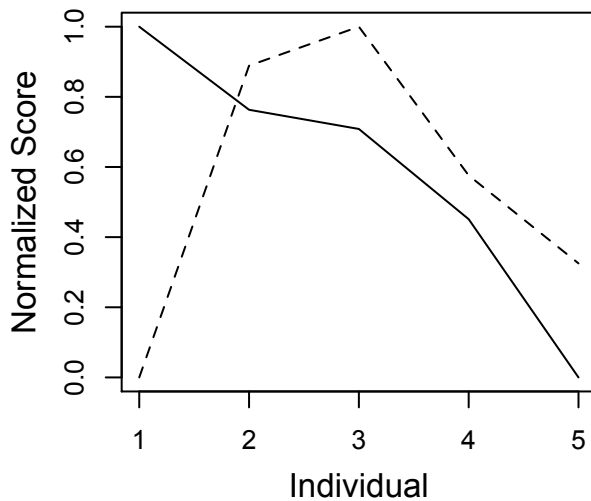**GL**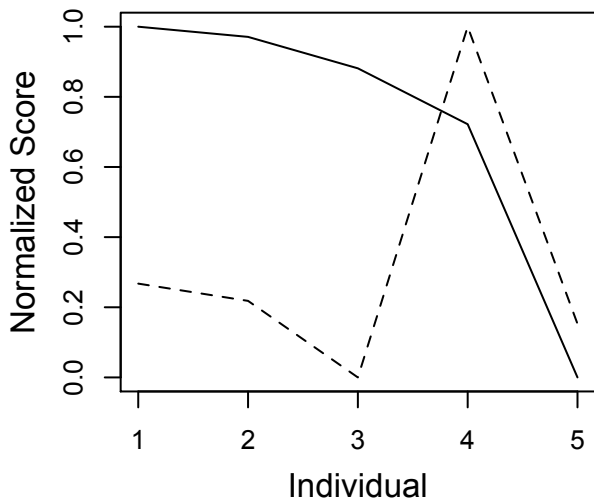 **$\beta$** 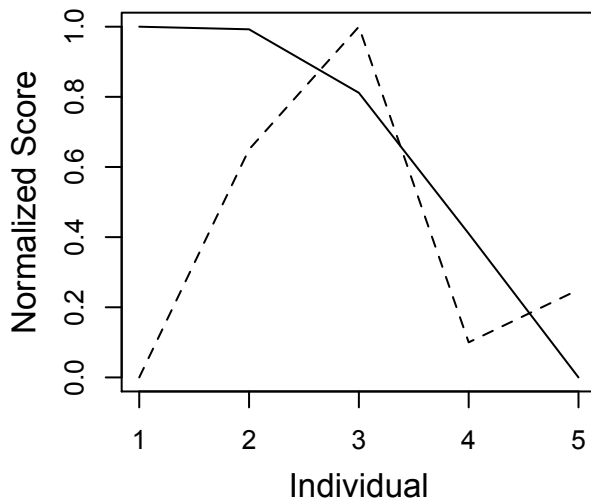

Supplement: Figure S12 — Each plot shows the (normalized) entropy scores and (normalized) algorithm scores for the four algorithms that do not correlate well with entropy on four artificial data sets constructed to show this lack of correlation. The solid lines are the entropy scores and the dashed lines are the algorithms. (PDF) [file pcbi.1003109.s012.pdf]
