# Supplementary material for: A Family of Algorithms for Computing Consensus about Node State from Network Data
Source: PLoS Comput Biol. 2013 Jul 18;9(7):e1003109. doi: 10.1371/journal.pcbi.1003109 (PMC3715438; doi:10.1371/journal.pcbi.1003109)
Supplement: Text S1 — The Supporting Information contains the following sections: 1. Sample entropy issues 2. Empirical evaluation of redistribution weights for Eigenvector Centrality 3. Extension of David's Score 4. Comparison of David's Score and Eigenvector Centrality 5. Sensitivity to source bias on transitive networks 6. Mathematical intuition for insensitivity to source biases 7. Computational complexity. (PDF) [file pcbi.1003109.s013.pdf]

Supporting Information

This is the Supporting Information for Brush, E., Krakauer, D.C., and Flack, J.C. "A Family of Algorithms for Computing Consensus about Node State from Network Data"

The Supporting Information contains the following sections:

1. Sample entropy issues
2. Empirical evaluation of redistribution weights for Eigenvector Centrality
3. Extension of David's Score
4. Comparison of David's Score and Eigenvector Centrality
5. Sensitivity to source bias on transitive networks
6. Mathematical intuition for insensitivity to source biases
7. Computational complexity

Sample entropy issues

We use Shannon entropy $H(\{p_i\}) = -\sum_i p_i \log(p_i)$ as a measure of the agreement among partners with respect to a target node.

Lacking knowledge of the true probabilities of a node receiving interactions (*e.g.* signals) from various partners, we use estimates of those probabilities to estimate the entropy of the true distribution. In other words, rather than using the true probability of node i receiving an interaction from node j we use the estimate $\frac{S_{ji}}{R_i}$ where S_{ji} is the number of interactions sent from j to i and R_i is the total number of interactions i receives. These estimates are used to form a naive estimate of the entropy of the probability distribution $H = -\sum_i \frac{S_{ji}}{R_i} \log(\frac{S_{ji}}{R_i})$. Whereas $\frac{S_{ji}}{R_i}$ is a good estimate of the true probabilities, a function (in our case, entropy) of the estimates of the probabilities is not in general a good estimate of the function of the true probabilities. In particular, this naive estimate is known to underestimate the true entropy of the distribution. The main problem with the naive estimate is that we may not observe an event with low probability so that our estimate of the probability of that event will be 0, spuriously decreasing the entropy of the distribution.

Two methods for improving this naive estimator have been proposed with the goal of estimating the true entropy of the underlying probability distribution, one by David Wolpert and David Wolf [1] (what we will call the Wolpert estimator) and another by Ilya Nemenman, Fariel Shafee, and William Bialek [2] (what we will call the Nemenman estimator). In a Bayesian framework, the a posteriori distribution of the entropy of a distribution can be written as a function of the observed data and the a priori distribution of the generative probabilities. This a posteriori probability is itself intractable, but Wolpert and Wolf calculate its moments [1]. The Wolpert estimator is the expected Shannon entropy of a distribution using observed numbers of the various possible events. This estimator is proved to minimize mean-squared error. For a given probability distribution, the naive estimator may on average be closer to the true entropy than the Wolpert estimator (where the average is taken over all possible observed counts resulting from that probability distribution). However, this benefit is negated by the much higher variance of the naive estimator as compared to the Wolpert estimator. A problem with the Wolpert estimator is that the variance of the a posteriori distribution of entropy values is quite low and therefore the a priori distribution of entropy values strongly affects the a posteriori expectation of the entropy. The Nemenman estimator circumvents this issue this by integrating over *a priori* distributions [2].

Despite the improvements made upon the naive estimate of entropy, we use the naive estimate for two main reasons. First, in the primate communication network, the individuals appear to make

relatively accurate estimates of their relative power. We are interested in how they are making these computations. The naive entropy estimate is itself a rather complicated function, but it is much more straightforward than the improved estimates. It is unlikely that the animals are performing calculations of even the naive entropy estimate, but they may be gauging general consensus in the entropy sense. In that case, the naive estimate will be close to an estimate of general consensus. Further, although it is the case that we as the observers have only partial data and limited sample sizes, this is also certainly true for the animals who will not have observed every signal and who have limited memory and hence only access to a finite sample size. The animals are not likely to be taking into account missing data when they make their calculations.

Additionally, the largest differences between the naive entropy estimate and the other entropy estimators occur for those nodes who have the fewest interactions (*e.g.* receive the fewest signals and, in some cases, only a single signal). To evaluate whether this is an issue in our data set we do the following. In the signaling network, we calculate the entropy for each node's distribution of signals received as using each of the three estimators– naive, Wolpert, and Nemenman. We then calculate the precent deviation from the naive estimator for both the Wolpert and Nemenman estimators and take the maximum of these errors to be the worst case error (Figure S7). For those individuals whose receiving distribution has a high naive entropy estimate, we see that the error is quite small. We know that power scores are most predictive for those individuals with the highest scores, i.e. those with highest entropy of signals received, and quite poor for those individuals with low scores. Therefore, the exact value of the entropy is most important for those individuals with high scores and we find that the naive estimator is quite good for those individuals. Because of the computational complexity of the other estimators and because the naive estimate performs quite well for those individuals whose scores are most important, we choose to use the naive estimator in our work rather than the improved estimators of Wolpert and Nemenman.

Empirical evaluation of redistribution weights for eigenvector centrality

We have the good fortune of having external data to which we can compare the output of Eigenvector Centrality. For each data set we analyze, we can determine the redistribution weight w that optimizes the predictive value of Eigenvector Centrality. Using linear regression, we ask which value of the weighting parameter gives a distribution of scores that best predicts our external data. As described in the main text, for the subordination signaling network there are three dependent variables: intervention cost, aggression used, and support solicited (see Methods for operational definitions). For the physicist collaboration network the dependent variable is the total size of grants awarded by the NSF. For the functional linkage network the dependent variable is competitive fitness. Figure S8 shows how the predictive value of eigenvector centrality changes as a function of the parameter values for each of the data sets.

In systems with interaction matrices but without additional data, we would need another way to choose the weight. We find that a good rule of thumb is to use the weight w that minimizes the skewness of the distribution of scores. By minimizing skewness, we are decreasing the highest score(s) and introducing variation into the lower tail of the distribution. This helps distinguish between lower valued nodes and makes Eigenvector Centrality more predictive of functional data where there is variation in the lower end of the distribution. Figure S9 shows the relationship between the skewness of the eigenvector centrality scores and the predictive value of the algorithm, for a range of redistribution weights.

In the primate communication network, the weight that minimizes skewness and maximizes predictive value is $w = .95$. In this system, we must increase the redistribution parameter to nearly 1 in order to sufficiently decrease the skewness of the distribution caused by having one node with a disproportionately high score. In the collaboration network, the redistribution weight that maximizes predictive value is $w = .95$ and the redistribution weight that minimizes skewness is $w = .6$. We use $w = .95$. However, in the absence of external data, using the rule of thumb of minimizing skewness would produce very similar scores. In the gene functional linkage network, we find that the redistribution weight that minimizes skewness and maximizes predictive value is $w = 0.05$. Even though this weight is quite small, it cannot be ignored. There are three connected components of the graph. Having a positive

redistribution weight facilitates a comparison between the components and avoids the problem of giving spuriously high Eigenvector Centrality scores to nodes in small connected components.

Extension of David's Score

In his original paper, David provides two ways to compute David's score, one of which we described in the main text and the second of which we present here [3]. Let $E = P + \frac{1}{2}I$ where I is the $n \times n$ identity matrix. Then

$$E^2 = \left(P + \frac{1}{2}I\right)^2 = P + P^2 + \frac{1}{4}I$$

and

$$(E^T)^2 = \left(P^T + \frac{1}{2}I\right)^2 = P^T + (P^T)^2 + \frac{1}{4}I$$

and the vector of power scores according to David's score is given by

$$s = \mathbf{1}^T \cdot (E^2 - (E^T)^2) = \mathbf{1}^T \cdot (P + P^2 - P^T - (P^T)^2).$$

As explained in the main text, David's score weights the probabilities that an individual will receive an interaction by the probabilities that its partners will receive interactions and the probability that an individual will send an interaction by the probabilities that its partners will send interactions. However, we could go further to consider the senders to the senders to the senders to the focal individual, and the receivers of the receivers of the receivers of the individual, continually going more and more levels through the network. We might, therefore, want to define a generalized David's score. We could define a generalized David's score using either of two methods:

$$s_n = \mathbf{1}^T \cdot (E^n - (E^T)^n) \text{ or } s_n = \mathbf{1}^T \left(\sum_{i=1}^{n} P^i - \sum_{i=1}^{n} (P^T)^i \right).$$

For $n = 2$, these definitions are identical, but for $n > 2$, these two generalizations are slightly different because of the expansion of E^n. They are, however, practically indistinguishable. The generalization using E is computationally easier to compute, so we will use this generalization. In Figure S10, we show the power scores for $n = 2, 5, 10, 15, 20$ and 25, normalized to range between -1 and 1 for easier comparison. On the subordination signaling network, the rank order of power scores computed using David's Score begins to converge at about $n = 15$. We can also see that as we increase n we lose the ability to distinguish between most individuals in the population, since one individual has maximum power, one individual has very low power, and all other individuals have scores close to 0. Because of the increased computational difficulty of David's score for higher n, the similarity in rankings produced by David's score for various n, and the inability of David's score to distinguish between individuals in the middle of the distribution for higher n, we only consider David's score for $n = 2$.

Comparison of David's score and Eigenvector Centrality

David's Score is closely related to Eigenvector Centrality. Both algorithms turn the interaction matrix into a matrix representing probabilities of interaction and then consider the probability of moving from node to node. There are, however, three differences between David's Score and Eigenvector Centrality:

1. Eigenvector Centrality requires a stochastic matrix where each column sums to 1, so that the elements of the matrix represent the probability of an node sending an interaction to any other given node. David's Score requires a matrix where elements [are] opposite each other across the diagonal, i.e. P_{ji} and P_{ij}, sum to 1, so that the elements of the matrix represent the probabilities with which each node sends an interaction to the other node in the pair.

2. Eigenvector Centrality calculates the probability of spending time at a node during an infinite random walk. David's Score is similar to calculating the probability of ending up at a node after a random walk of length 2. Extensions of David's Score can be constructed by increasing the length of the walk, which we consider in Section Extension of David's Score .

3. With Eigenvector Centrality, in addition to the probabilities of moving from node to node given by the interaction matrix there are also probabilities of moving from node to node given by redistribution probabilities. In the random walk in David's Score there is no redistribution.

Sensitivity of eigenvector centrality on transitive networks

In our analysis of sensitivity to source bias, we found that eigenvector centrality is significantly less sensitive on the primate communication network. This is in part to due to the high transitivity of the communication network. Consider a target node with an initially high eigenvector centrality score. The high transitivity means that there is in general a negative relationship between the centrality of a node and the number of animals signaled by that node. Therefore, one of two things can happen when we introduce strong source bias, i.e. when all of the target node's signals come from one partner node. First, the partner node could also have high power, which would then be conferred upon the receiver. Second, the partner node could have low power but signal many other nodes so that in the shuffled matrix the partner sends many signals to the target and relatively few signals to his other receivers, decreasing the power of those many other animals and leaving the receiver with a relatively high score. Thus, on a highly transitive network, it is difficult to reduce the rank of a high ranking target node by introducing source bias. Similarly, since no high centrality node ever signals a low centrality node, introducing source bias will not greatly increase the scores of low power nodes. The fact that the ranks do not change when we introduce source bias, therefore, does not mean that eigenvector centrality cannot detect manipulations of the the input matrix. Rather, it reflects the fact that eigenvector centrality correctly identified as highly ranked those nodes that received many signals from other highly ranked nodes. This makes eigenvector centrality a good measure of consensus on transitive networks.

If we reduce the transitivity in the communication network, we find that the sensitivity of eigenvector centrality increases (Figure S11). We construct a random matrix where each node has the same in-degree and out-degree as in the communication network but where the edges are randomly assigned. This random matrix therefore has the same in-degree and out-degree as the the original communication network, but the correlation between centrality and number of partner nodes is decreased and transitivity is reduced. On this matrix, the second or third order connections are less informative about first order connections, and eigenvector centrality can detect disruptions to the first order connections.

Mathematical intuition for insensitivity to source biases

For four of our algorithms, Eigenvector Centrality C, David's Score, the graph Laplacian GL, and the Borda count β_i, we create a fictitious interaction matrix in which nodes receiving many interactions receive all or most of the interactions from one or a few partner nodes. Using the matrix constructed for each algorithm, we calculate the Shannon entropy of the probability distribution of interactions received by each node and the consensus scores using the algorithm. We ask how correlated the algorithm's distribution of consensus scores is with the distribution of Shannon entropies. This analysis only evaluates whether it is possible for an algorithm to be poorly correlated with Shannon entropy, and hence is a cruder analysis than our shuffling analysis in the main text. Figure S12 shows, for each algorithm, the entropy scores (solid lines) and the algorithm scores (dashed lines) on the artificial data matrix designed for that algorithm, where both set of scores have been linearly normalized to be between 0 and 1. These plots show visually how different the algorithms can be from entropy scores.

The following artificial signaling matrix represents a situation in which entropy and Eigenvector Centrality disagree (Kendall's $\tau = 0.316$, $p = .61335$).

$$M = \begin{pmatrix} 0 & 0 & 0 & 0 & 500 \\ 1 & 0 & 0 & 0 & 0 \\ 1 & 5 & 0 & 0 & 0 \\ 1 & 10 & 1 & 0 & 0 \\ 1 & 15 & 1 & 100 & 0 \end{pmatrix}$$

where M_{ij} is the number of signals individual i sends to individual j. C_i gives high scores to individuals

who receive a high proportion of other individuals' signals, and is insensitive to the absolute number of signals received and how closely the numbers of signals emitted by different individuals agree. On this data set, those individuals who receive similar numbers of signals from their signalers receive only a low proportion of the signals emitted by those individuals (and conversely), so C_i and entropy tend to disagree. For example, individual 4 has an entropy score of 0 since he only receives signals from one other individual. However, since individual 4 receives a high proportion of individual 5's emitted signals and 5 receives many signals node 4 has a high centrality score.

The following artificial signaling matrix represents a situation in which entropy and David's Score disagree (Kendall's $\tau = 0$, $p = 1$).

$$M = \begin{pmatrix} 0 & 30 & 10 & 50 & 70 \\ 1 & 0 & 40 & 6 & 0 \\ 1 & 40 & 0 & 0 & 0 \\ 1 & 60 & 10 & 0 & 0 \\ 1 & 0 & 2 & 6 & 0 \end{pmatrix}$$

David's score, like C_i, only uses information about the proportion of signals emitted between each pair, and not the agreement in absolute number of signals received from various individuals. In this data set, individuals about whom the population agrees send many more signals than they receive (and conversely), so entropy and David's score are not correlated. For instance, individual 1 has the highest entropy score because the whole population sends it the same number of signals. However, it signals much more to the other individuals than they signal to it, giving him a low David's score.

The following artificial signaling matrix represents a situation in which entropy and the graph Laplacian disagree (Kendall's $\tau = 0.2$, $p = 0.8065$).

$$M = \begin{pmatrix} 0 & 4 & 3 & 0 & 0 \\ 5 & 0 & 7 & 0 & 0 \\ 5 & 6 & 0 & 2 & 0 \\ 0 & 0 & 0 & 0 & 0 \\ 0 & 0 & 0 & 8 & 0 \end{pmatrix}$$

The graph Laplacian, GL_i, is not designed to give high scores to those individuals who receive a similar number of signals from many signalers (or who receive signals from many signalers). The algorithm will give a high score to an individual who receives many signals from individuals who themselves have lower entropy scores. In this data set, individuals about whom there is consensus as measured by entropy tend to interact with individuals with similar entropy scores, resulting in a poor correlation between entropy and the graph Laplacian. For example, Individual 1 has the highest entropy score because his two signalers send the same number of signalers. However, its entropy score is quite close to the entropy scores of individuals 2 and 3, from whom it receives signals and to whom he signals. The nearly equal number of signals it receives and sends from those individuals and the closeness of their entropy scores means that there is not very much flow to node 1, giving individual 1 a low graph Laplacian score.

The following artificial signaling matrix represents a situation in which entropy and the Borda count disagree (Kendall's $\tau = -0.2$, $p = 0.8065$).

$$M = \begin{pmatrix} 0 & 10 & 20 & 5 & 5 \\ 10 & 0 & 30 & 15 & 15 \\ 10 & 11 & 0 & 10 & 11 \\ 10 & 11 & 25 & 0 & 5 \\ 10 & 11 & 35 & 10 & 0 \end{pmatrix}$$

The Borda count, like C_i and David's score, does not take into account the absolute number of signals received nor their agreement across signalers. On this data set, individuals who receive similar numbers of signals from the population are also given low ranks by the rest of the population, resulting in a negative correlation between the Borda count and entropy. For example, while all individuals agree that they should send 10 signals to individual 1, they tend to send fewer signals to individual 1 than to other members of the population. Therefore, individual 1 has the highest entropy score but the lowest Borda count.

Computational complexity

In deriving each algorithm and the relationships between them, we have focused on calculating a consensus score for each node in the network. However, the consensus score of each node is in many case only meaningful in comparison to the distribution of consensus scores of the rest of the network. This means that we need to consider the computational complexity of each algorithm with respect to the calculation of relative scores, in addition to absolute scores.

As described in the main text, there are two main procedures we follow to calculate any algorithm: first we transform the interaction matrix into a new matrix and then we perform (sometimes repeated) matrix operations on these matrices. The complexity of transforming the interaction matrix depends on the calculations required for the transformation. For instance, the matrix of binary interactions, B, is presumably easier to calculate and store than the matrix $\widehat{S}$ used for Shannon entropy. Additionally, we suspect that the complexity of an algorithm increases with the number matrix operations required to calculate the consensus scores. For instance, if $\widehat{W}$ and P, the matrices used for Eigenvector Centrality and David's score, are comparably complex to calculate and store, Eigenvector Centrality should be more complex to compute since it requires that $\widehat{W}$ be multiplied by itself many times whereas we only have to square P to compute David's score.

For the uniformity-based algorithms, the complexity of the calculation will scale linearly with the number of nodes. If there are n nodes in the network, calculating the consensus scores for every node in the network will be n times as computationally complex. This is because an nodes's score is simply a function of that node's receiving vector. On the other hand, it seems likely that the complexity of the diffusion-based algorithms scale sublinearly with the number of individuals whose scores we want so that calculating the consensus scores for every node in the network will involve fewer than n times the number of calculations for each consensus score individually.

If we imagine trying to calculate a target node's Eigenvector Centrality by actually performing the random walk, we would have to spend time at the other nodes in the network and would therefore get an estimate of the time spent at other nodes in the process of calculating the target node's score. We would not have to perform the random walk calculation n times in order to determine the scores for n nodes, as we could keep track of the time spent at all nodes during one random walk.

The complexity of the Borda count will also scale sublinearly with the number of nodes whose scores we want to calculate for similar reasons. In evaluating a given individual's Borda Count score, we have to determine how each partner ranks that the target node, which involves looking at all of the interactions sent by each partner. In trying to determine where a given node sits within a ranking, we have to find the whole ranking and, having done this, calculating the Borda count for all nodes is not much harder than calculating it for a focal individual.

This suggests that we can divide the set of algorithms into two sets. One set consists of the uniformity based algorithms that are less complex to calculate for one node but have a "mathematical complexity" that scales linearly with the number of nodes whose scores we are interested in. The second set consists of the diffusion and count based algorithms that are, we argue, more complex to calculate for one node but which have a "mathematical complexity" that scales sublinearly with the number of nodes whose scores we are calculating. Even if we could quantify the complexity of the algorithms, we would have to decide whether to compare the computations required for calculating one node's score or the whole population's. The difference in scaling with number of nodes makes comparing algorithms from the different sets difficult.

References

[1] Wolpert DH, Wolf DR (1995) Estimating functions of probability distributions from a finite set of samples. Physical Review E 52: 6841-6854.

[2] Nemenman I, Shafee F, Bialek W (2002) Entropy and inference, revisited. arXiv .

[3] David HA (1987) Ranking from unbalanced paired-comparison data. Biometrika 74: 432-436.

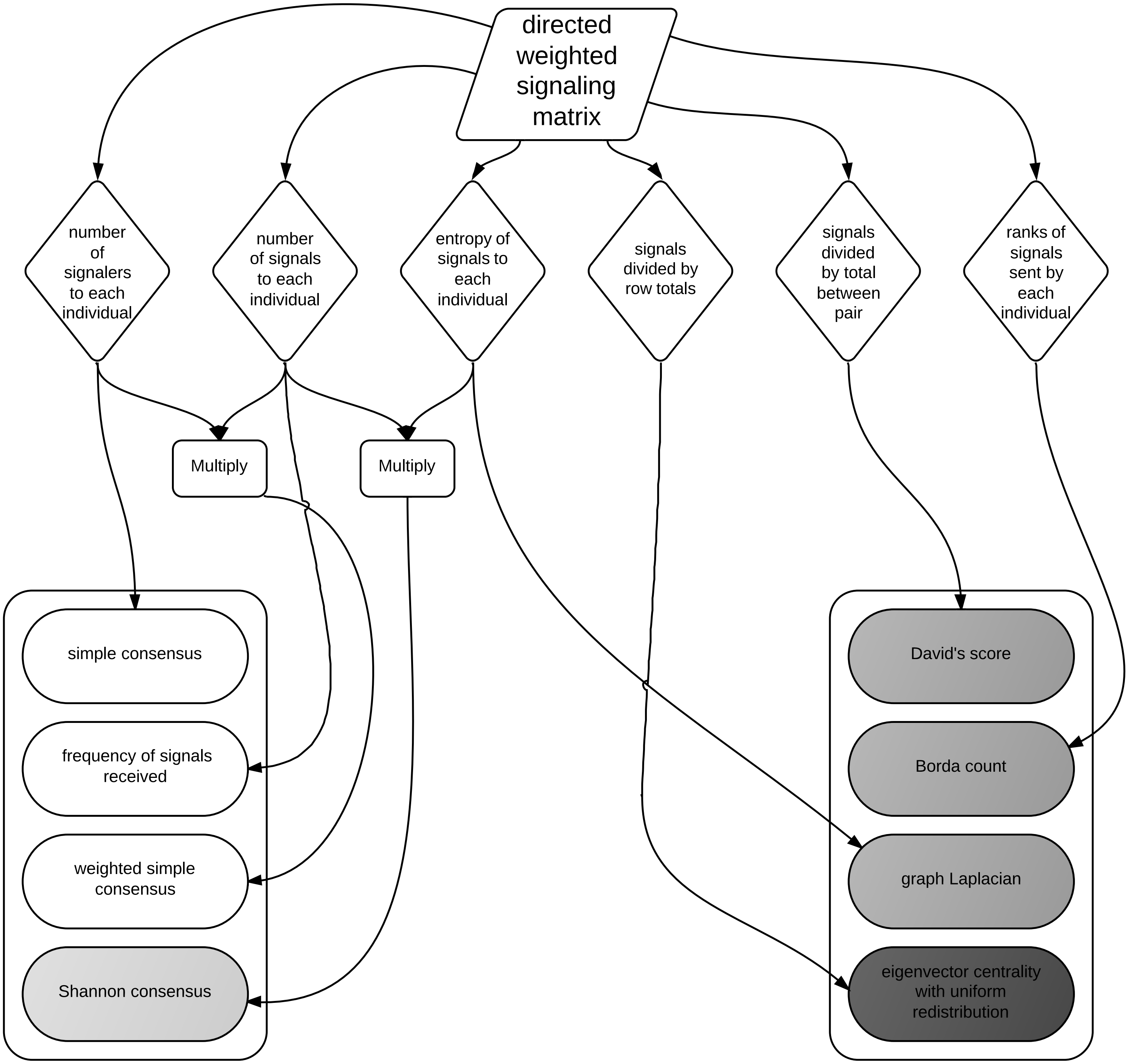
directed weighted signaling matrix
number of signalers to each individual
number of signals to each individual
entropy of signals to each individual
signals divided by row totals
signals divided by total between pair
ranks of signals sent by each individual
Multiply
Multiply
simple consensus
frequency of signals received
weighted simple consensus
Shannon consensus
David's score
Borda count
graph Laplacian
eigenvector centrality with uniform redistribution

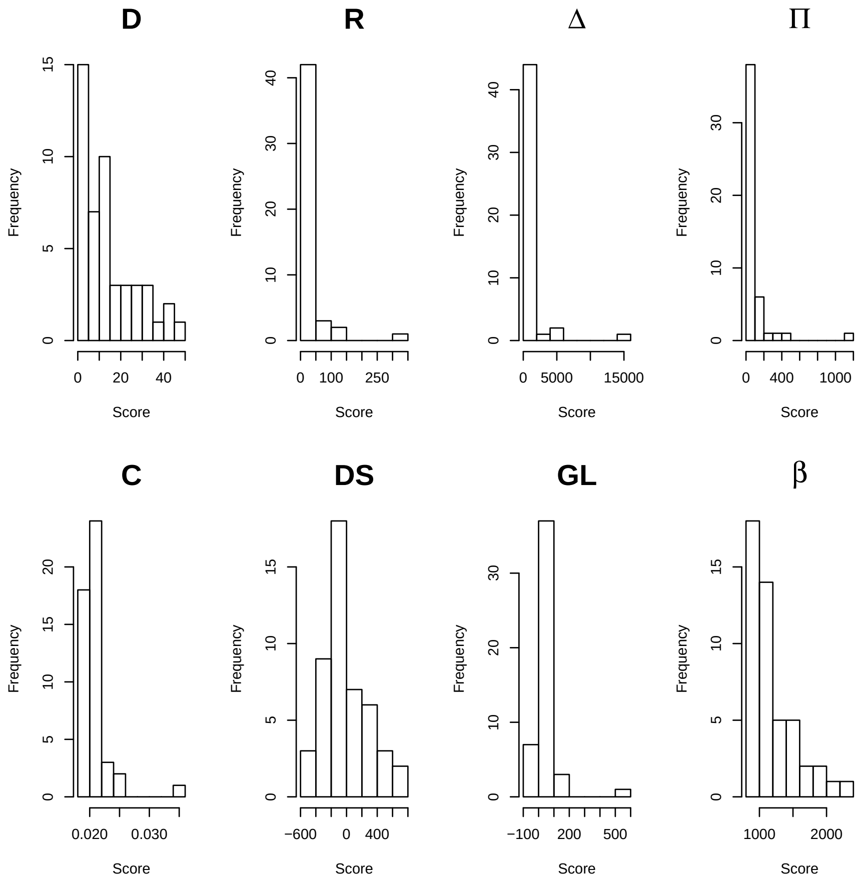

Figure S2. The distribution of the scores resulting from each algorithm as applied to the subordination signaling network.

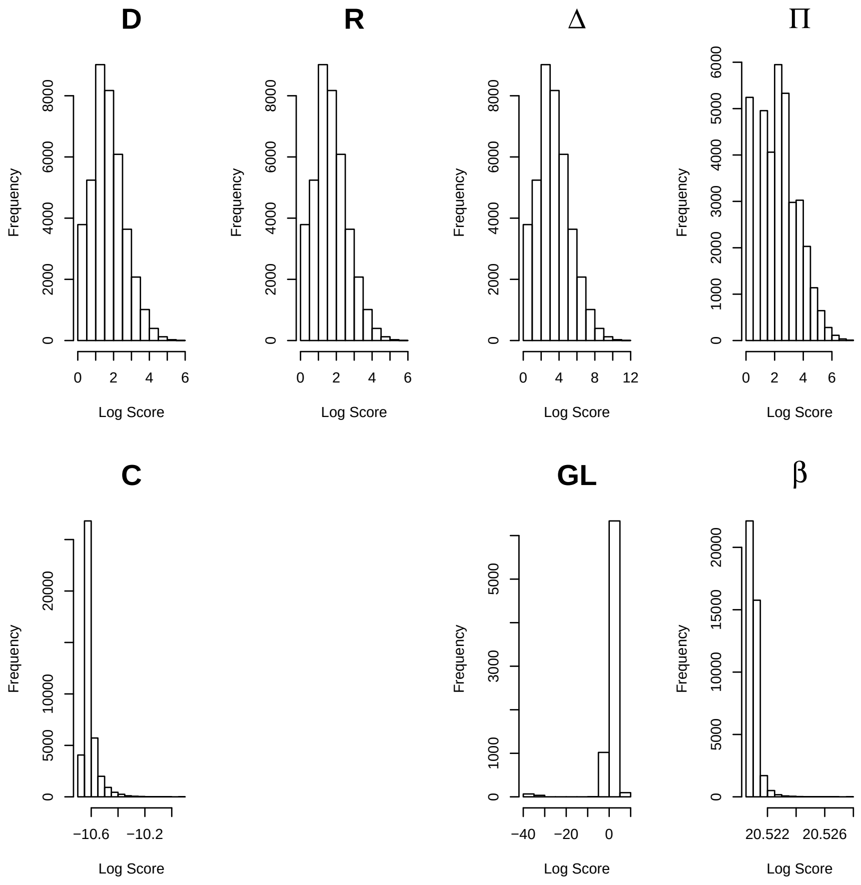

Figure S3. The distribution of the logarithm of the scores resulting from each algorithm as applied to the physicist collaboration network.

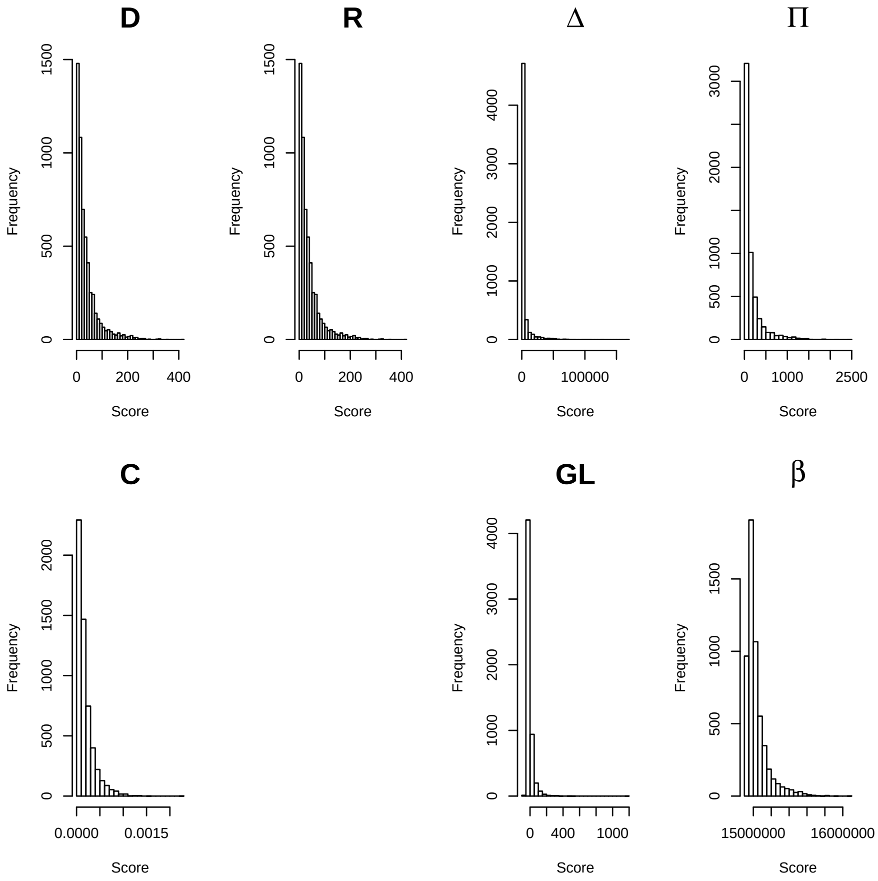

Figure S4. The distribution of the scores resulting from each algorithm as applied to the yeast functional linkage network.

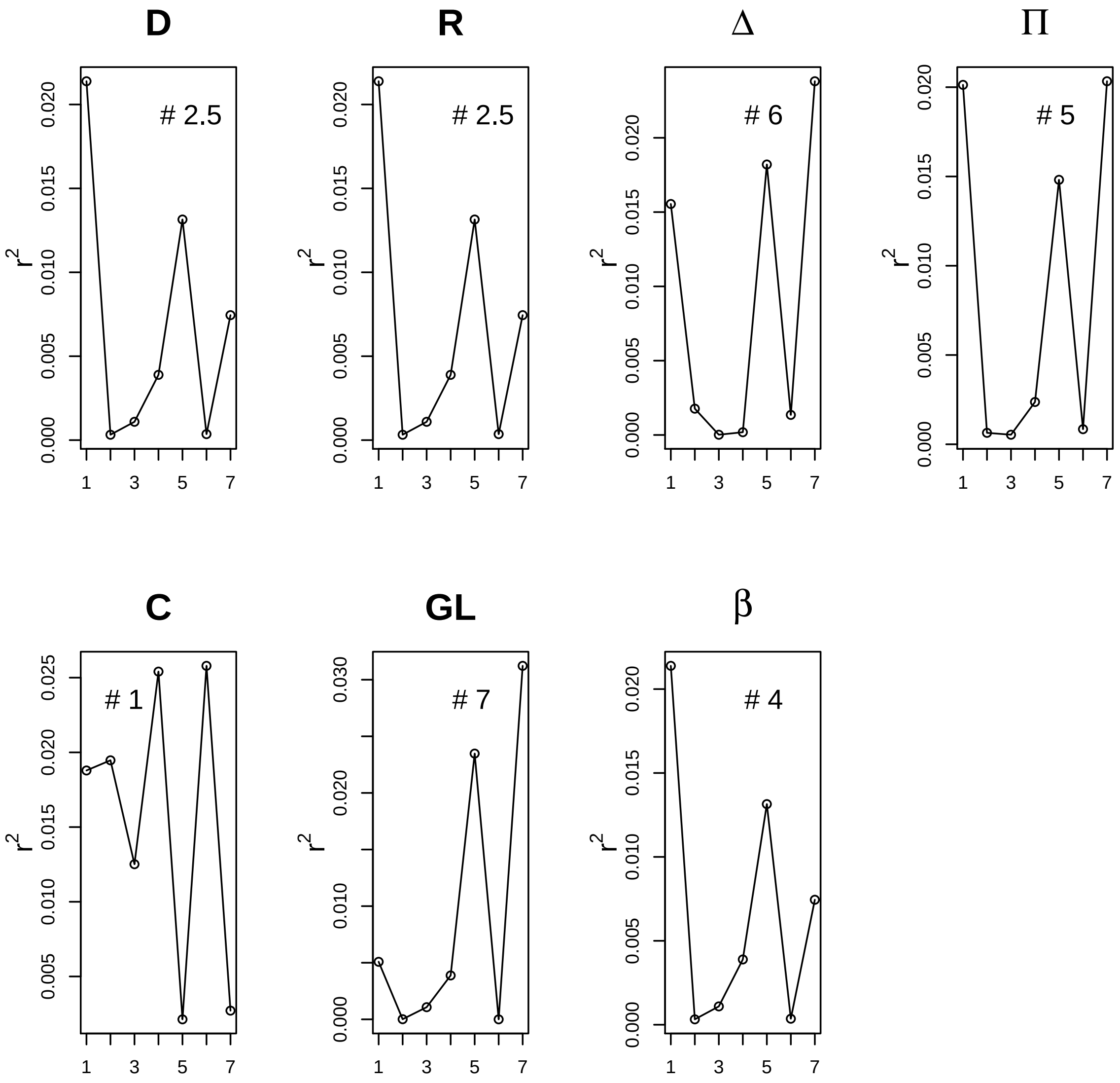

Figure S5. This figure shows for the physicist collaboration network the fit of each algorithm to the functional data. The x-axis indicates which subset of nodes are being considered– 1 is the top quartile, 2 is the top half, 3 is the top three quartiles, 4 is all nodes, 5 is the bottom three quartiles, 6 is the bottom half, and 7 is the bottom quartile– where the quartiles may vary from algorithm to algorithm (see Sec. Prediction heterogeneity). The number in the each plot indicates the rank of each algorithm with respect to its performance predicting the functional data.

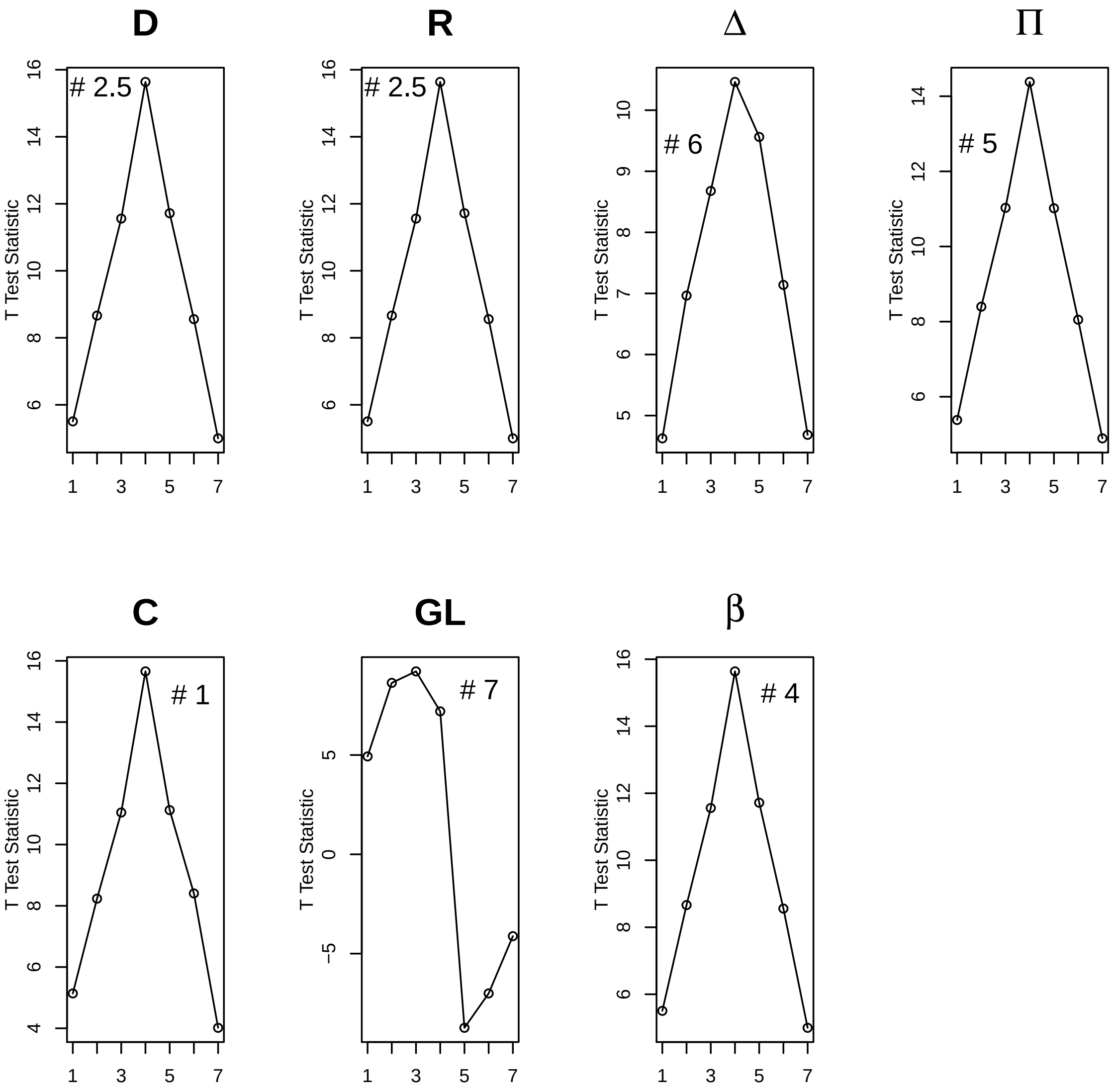

Figure S6. This figure shows for the functional linkage network of genes the fit of each algorithm to the functional data. The x-axis indicates which subset of nodes are being considered– 1 is the top quartile, 2 is the top half, 3 is the top three quartiles, 4 is all nodes, 5 is the bottom three quartiles, 6 is the bottom half, and 7 is the bottom quartile– where the quartiles may vary from algorithm to algorithm (see Sec. Prediction heterogeneity). The number in the each plot indicates the rank of each algorithm with respect to its performance predicting the functional data.

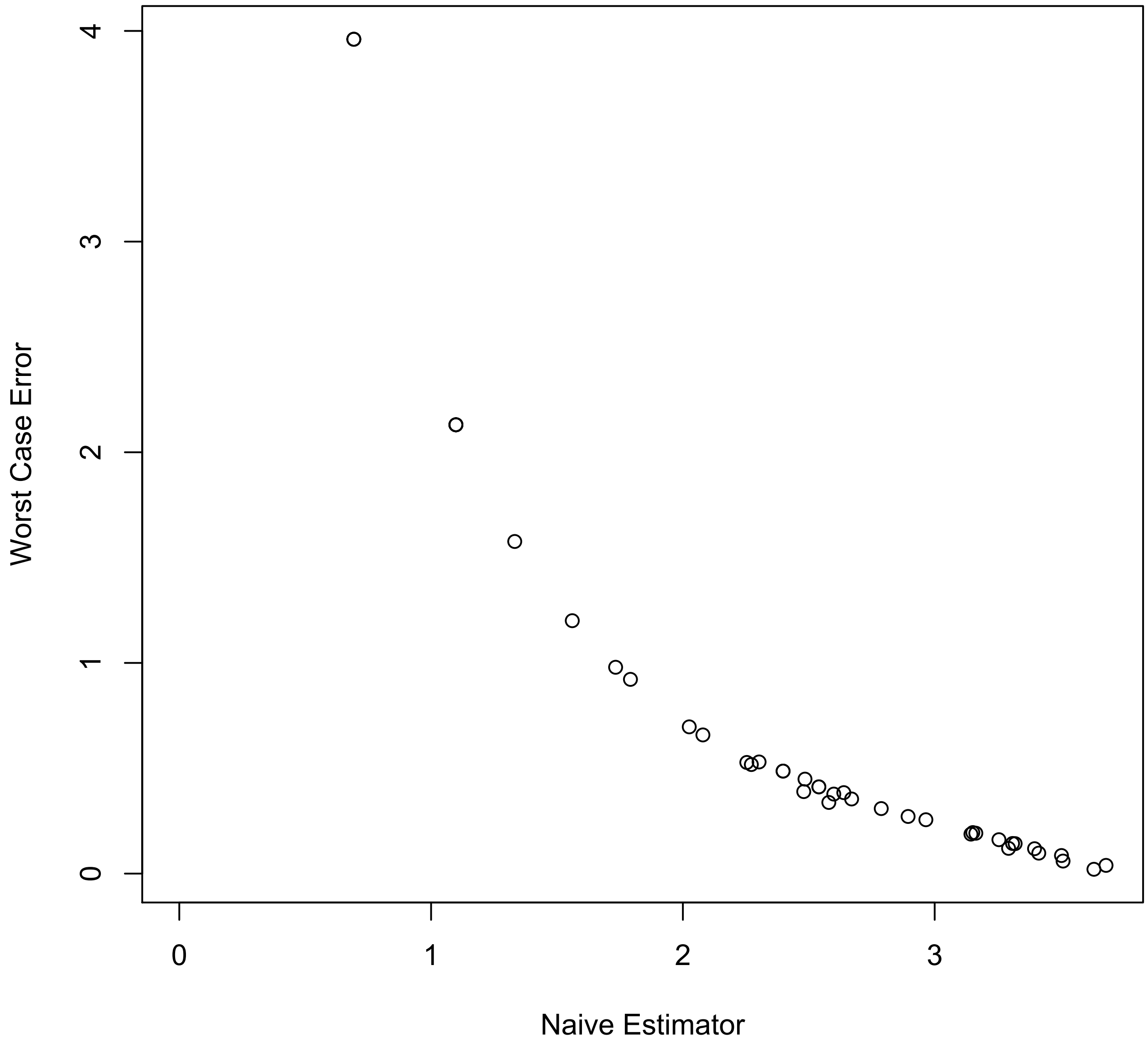

Figure S7. The worst case error of the naive entropy estimator as a function of the naive estimator. Each data point represents the naive entropy estimate and worst case error of one individual's receiving distribution. Figure was created by Simon DeDeo, 2011.

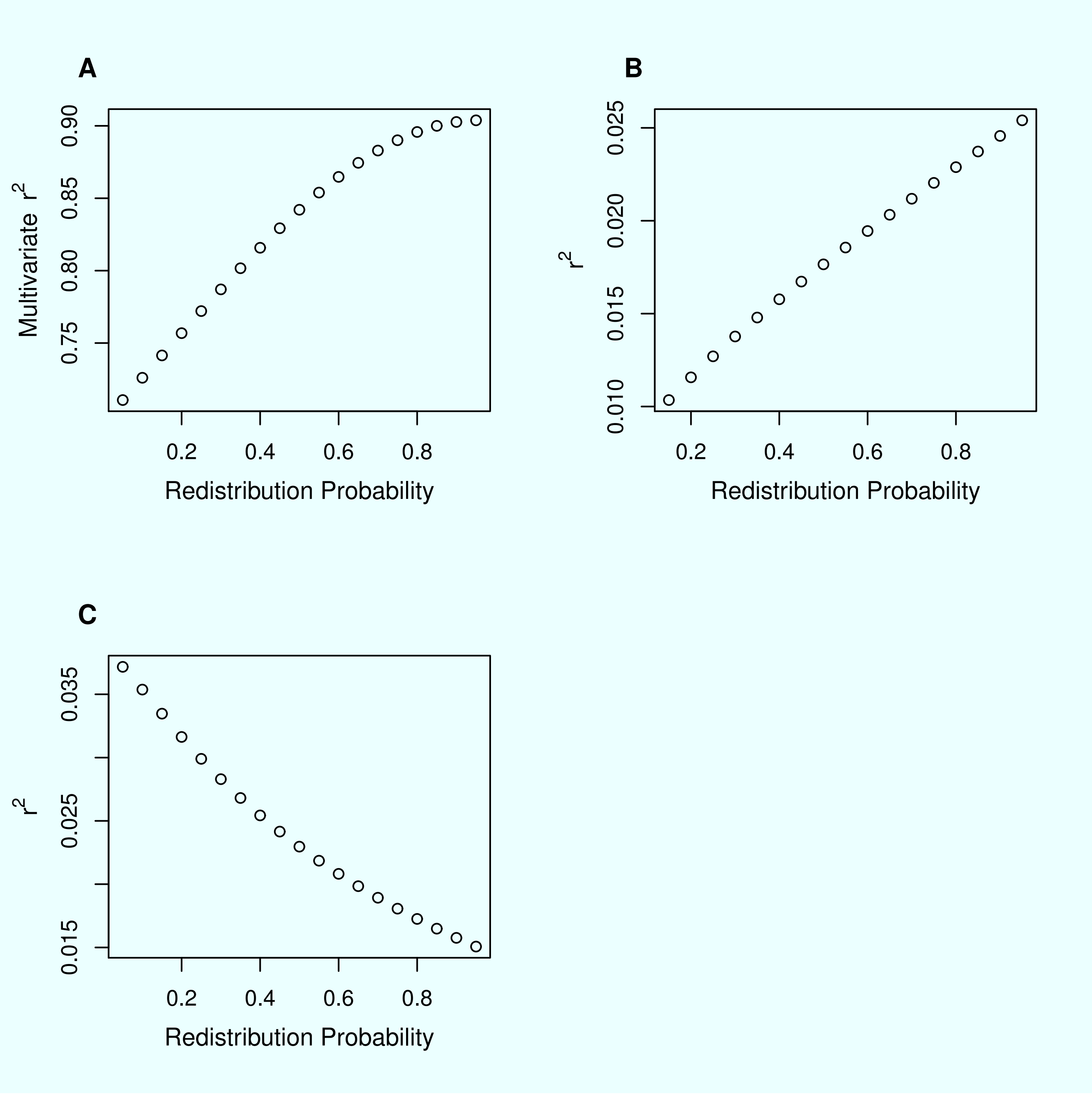

Figure S8. This figure shows how predictive value of eigenvector centrality changes as a function of the redistribution parameter. **A**. shows how the r^2 value from a multivariate regression of eigenvector centrality on the subordination signaling network against the three external data setsdepends on the redistribution probability. **B**. shows how the r^2 value from a regression of eigenvector centrality on the physicist collaboration network against the external data depends on the redistribution probability. **C**. shows how the r^2 value from a regression of eigenvector centrality on the functional linkage network against competitive fitness depends on the redistribution probability. In the subordination signaling network and the physicist collaboration network, eigenvector centrality is most predictive when the redistribution probability is about .95. In the gene interaction network, eigenvector centrality is most predictive when the redistribution probability is about 0.05.

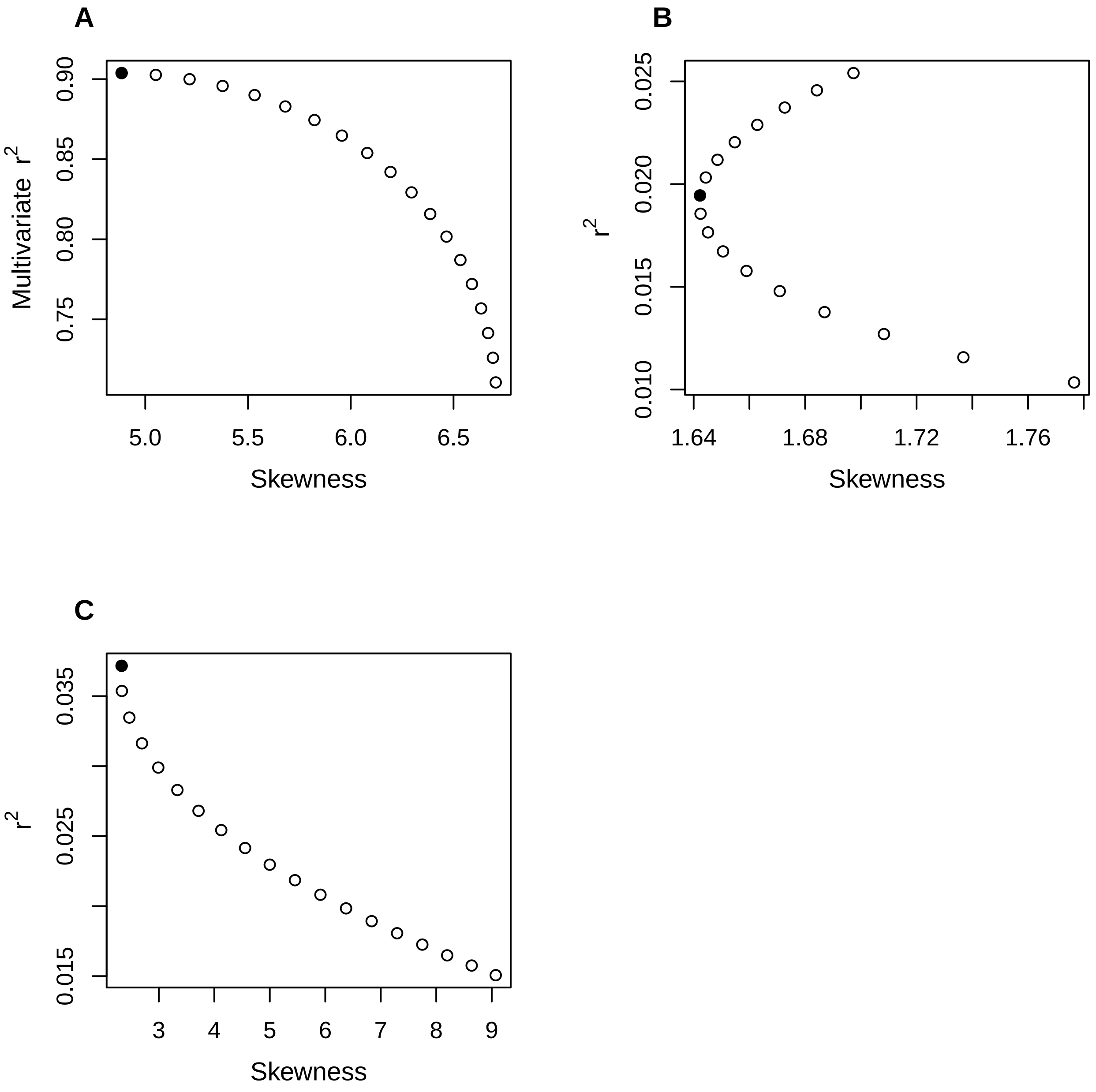

Figure S9. This figure shows how predictive value and skewness of eigenvector centrality scores are related. **A**. shows how the r^2 value from a multivariate regression of eigenvector centrality on the primate communication network against the external data depends on the skewness of the scores. **B**. shows how the r^2 value from a regression of eigenvector centrality on the physicist collaboration network against the external data depends on the skewness of the scores. **C**. shows how the r^2 value from a regression of eigenvector centrality on the functional linkage network against competitive fitness depends on the skewness of the scores. In the primate communication network, the redistribution weight that minimizes skewness is .95. In the collaboration network, the weight that minimizes skewness is .6. In the gene interaction network, the redistribution weight that minimizes skewness .05. These points are indicated in solid circles.

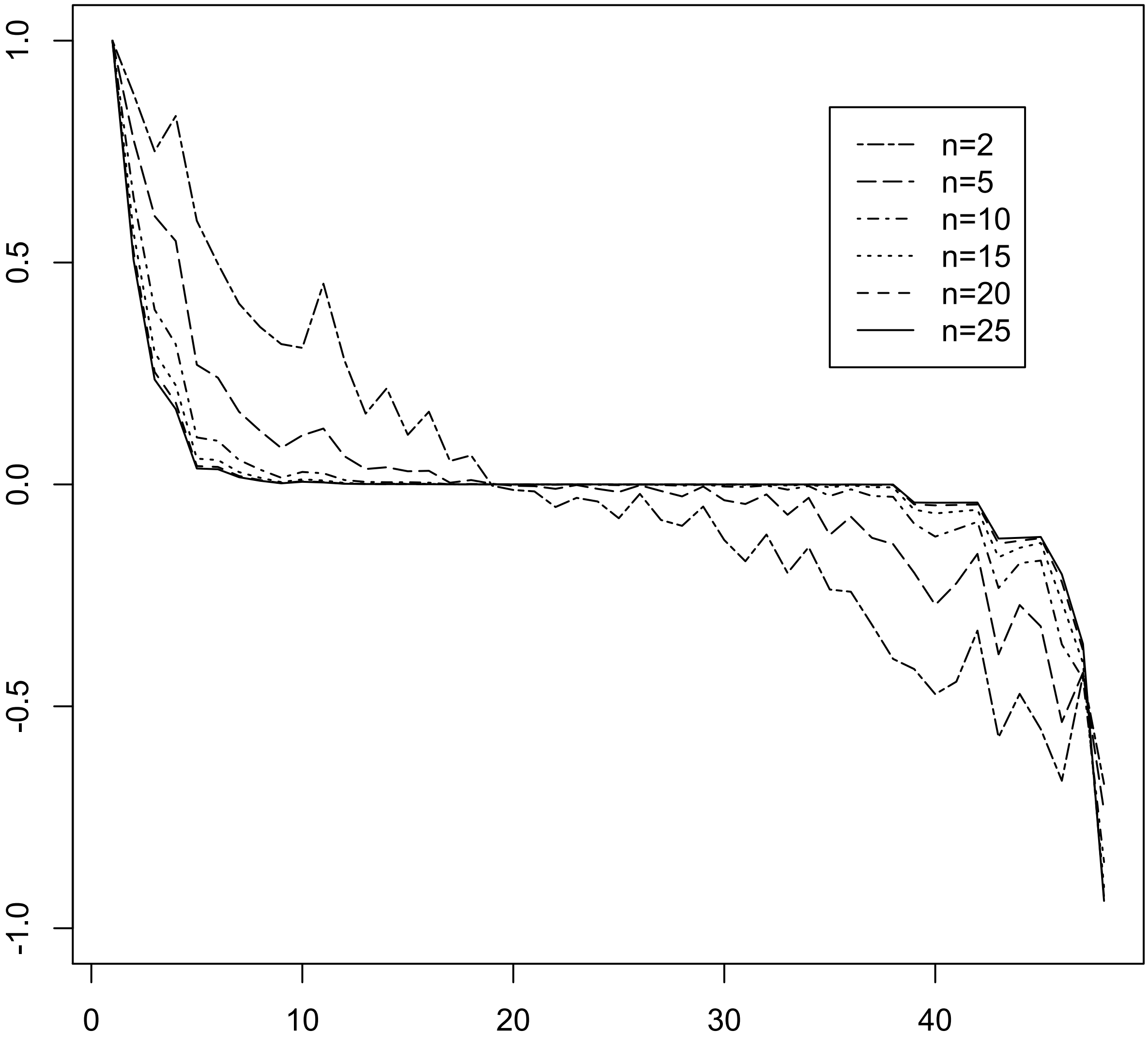

Figure S10. This figure shows the effects of varying the length of the random walk, n, on the power scores computed using David's Score. The y axis shows David's score for each individual, normalized so that the highest score is 1 and the lowest score is -1. Each line corresponds to the generalization of David's score for different n, as specified in the Figure legend. The plot shows that at about $n = 15$ the rank orders begin to converge.

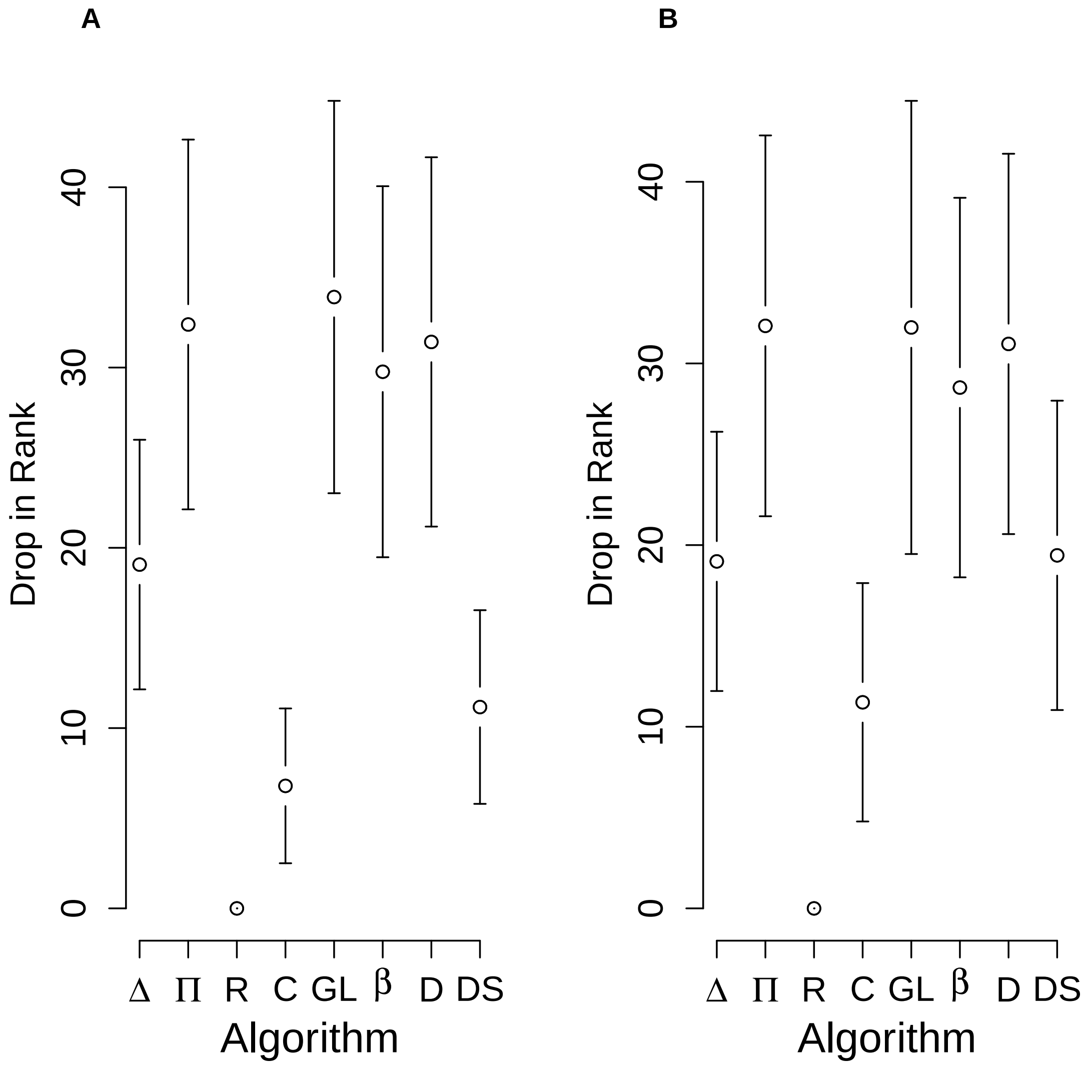

Figure S11. This figure shows show the sensitivity of each algorithm to source bias in a shuffled primate communication matrix. For each algorithm, we report the drop in rank induced when a node receives all of its edges from one of its neighbors. The point shows the mean correlation and the bars show plus or minus one standard deviation. The algorithms are ordered from left to right by their predictive power for the primate communication network. **A**. Primate communication network. **B**. We remove the transitivity in the primate communication network by constructing a random network where each node has the same in-degree and out-degree as in the primate communication network. On this network, eigenvector centrality is no longer significantly less sensitive than the other algorithms.

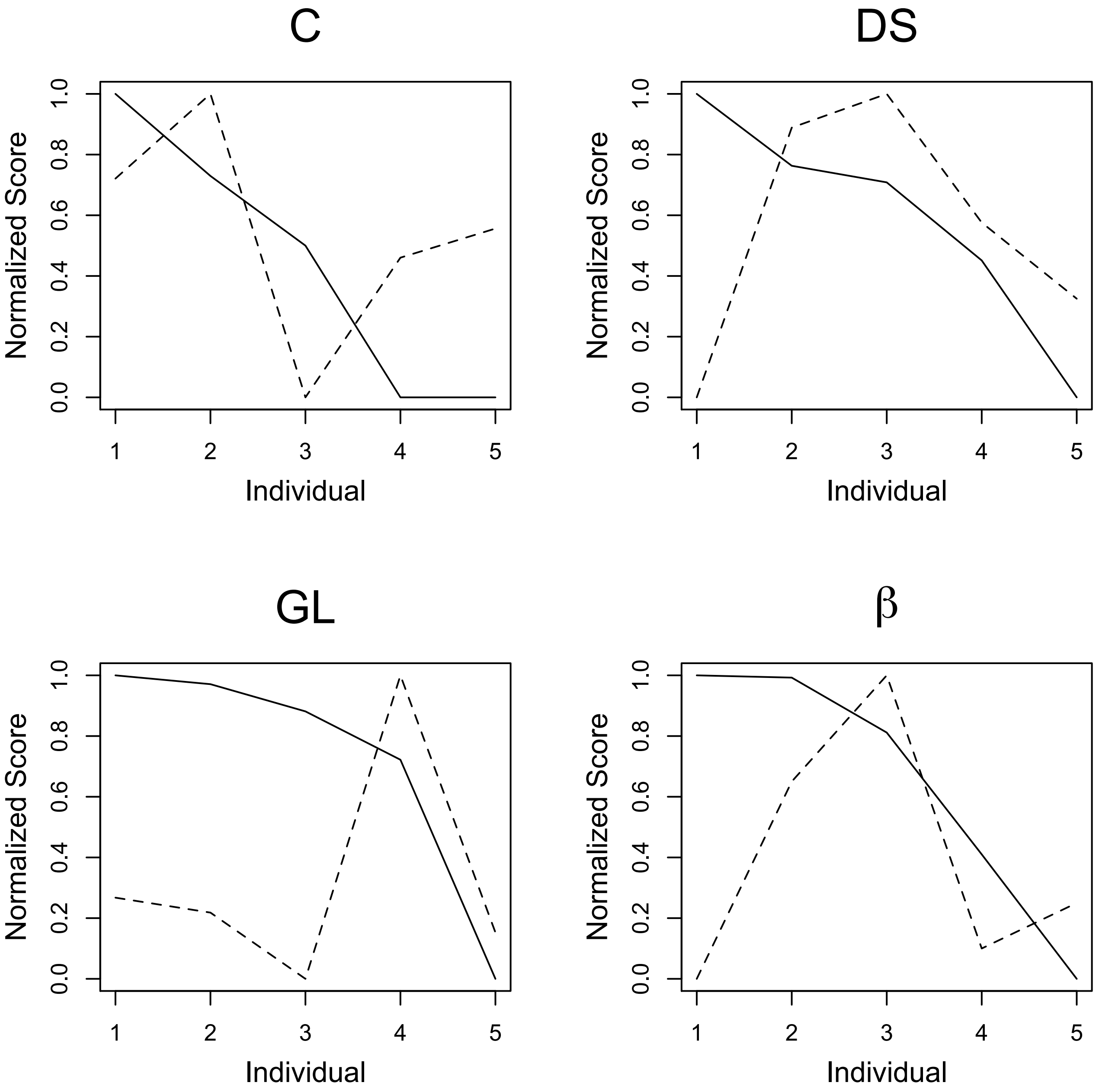

Figure S12. Each plot shows the (normalized) entropy scores and (normalized) algorithm scores for the four algorithms that do not correlate well with entropy on four artificial data sets constructed to show this lack of correlation. The solid lines are the entropy scores and the dashed lines are the algorithms.

Table S1. Table of Kendall correlations between algorithms.

(a). Subordination signaling network.

	H	R	D	Δ	Π	C	DS	GL	β
H	1	0.94	0.98	0.95	0.95	0.93	0.79	0.87	0.94
R	0.94	1	0.97	0.99	0.99	0.98	0.78	0.89	0.98
D	0.98	0.97	1	0.98	0.97	0.96	0.80	0.88	0.97
Δ	0.95	0.99	0.98	1	0.99	0.97	0.79	0.88	0.99
Π	0.95	0.99	0.97	0.99	1	0.97	0.79	0.89	0.99
C	0.93	0.98	0.96	0.97	0.97	1	0.79	0.87	0.98
DS	0.79	0.78	0.80	0.79	0.79	0.79	1	0.70	0.79
GL	0.87	0.89	0.88	0.88	0.89	0.87	0.70	1	0.88
β	0.94	0.98	0.97	0.99	0.99	0.98	0.79	0.88	1

(b). Physicist collaboration network.

	H	R	D	Δ	Π	C	GL	β
H	1	1	1	1	1	0.51	0.05	1
R	1	1	1	1	1	0.51	0.04	1
D	1	1	1	1	1	0.51	0.04	1
Δ	1	1	1	1	1	0.51	0.04	1
Π	1	1	1	1	1	0.51	0.05	1
C	0.51	0.51	0.51	0.51	0.51	1	0.50	0.51
GL	0.05	0.04	0.04	0.04	0.05	0.50	1	0.04
β	1	1	1	1	1	0.51	0.04	1

(c). Gene interaction network.

	H	R	D	Δ	Π	C	GL	β
H	1	1	1	1	1	0.96	0.20	1
R	1	1	1	1	1	0.96	0.20	1
D	1	1	1	1	1	0.96	0.20	1
Δ	1	1	1	1	1	0.96	0.20	1
Π	1	1	1	1	1	0.96	0.20	1
C	0.96	0.96	0.96	0.96	0.96	1	0.25	0.96
GL	0.20	0.20	0.20	0.20	0.20	0.25	1	0.20
β	1	1	1	1	1	0.96	0.20	1
